# IL-17 signaling protects against *Helicobacter pylori-*induced gastric cancer

**DOI:** 10.1080/19490976.2024.2430421

**Published:** 2024-11-26

**Authors:** Lee C. Brackman, Matthew S. Jung, Emily H. Green, Nikhita Joshi, Frank L. Revetta, Mark S. McClain, Nicholas O. Markham, M. Blanca Piazuelo, Holly M. Scott Algood

**Affiliations:** a Department of Medicine, Division of Infectious Disease, Vanderbilt University School of Medicine, Nashville, TN, USA; b Department of Pathology, Microbiology and Immunology, Vanderbilt University School of Medicine, Nashville, TN, USA; c Vanderbilt Institute of Infection, Immunity, and Inflammation (VI4), Vanderbilt University Medical Center, Nashville, TN, USA; d Tennessee Valley Healthcare System, Department of Veterans Affairs, Nashville, TN, USA; e School of Biological Sciences, Vanderbilt University, Nashville, TN, USA; f Department of Medicine, Division of Gastroenterology, Vanderbilt University School of Medicine, Nashville, TN, USA

**Keywords:** Interleukin 17, gastritis, Helicobacter pylori, gastric cancer

## Abstract

*Helicobacter pylori* infection is the predominant risk factor for the development of gastric cancer. Risk is enhanced by specific *H. pylori* virulence factors, diet, and the inflammatory response. Chronic activation of T helper (Th) 1 and Th17 pathways contributes to prolonged inflammation; yet, higher expression of IL-17 receptor (IL-17RA) is a favorable prognostic marker for survival after gastric cancer diagnosis. The protective impact of IL-17RA signaling is not understood. To investigate if IL-17RA signaling protects during *H. pylori-*induced carcinogenesis, the transgenic *InsGAS*^*tg/tg*^ mouse, which is prone to *H. pylori*-induced gastric cancer, was utilized. *InsGAS*^*tg/tg*^ mice and *InsGAS*^*tg/tg*^*Il17ra*^*-/-*^ mice were infected with a cag type 4 secretion system (T4SS) positive *H. pylori* strain for up to 6 months. Six weeks post-infection, IL-17RA deficiency led to increased bacterial burden, increased gastritis, and development of lymphoid follicles. Increased inflammation was associated with heightened cellular proliferation and earlier loss of parietal and chief cells in *InsGAS*^*tg/tg*^*Il17ra*^*-/-*^ mice. Gastric cancers developed more frequently by 3- and 6-months post-infection in *H. pylori*-infected *InsGAS*^*tg/tg*^*Il17ra*^*-/-*^ mice compared to *InsGAS*^*tg/tg*^ mice. Chronic inflammation was exacerbated with IL-17RA deficiency, characterized by elevated Th1/Th17 cytokines, increased B cell infiltration, and enhanced IgA production, despite reduced expression of the polymeric immunoglobulin receptor. Further, paragastric lymph nodes of *InsGAS*^*tg/tg*^*Il17ra*^*-/-*^ mice were enlarged relative to controls and displayed altered gene expression profiles. Increased inflammation was accompanied by a significant increase in *Cybb* expression, which encodes NADPH oxidase 2, suggesting that increased oxidative damage may occur in the absence of IL-17RA. Further, there is increased phosphorylation of histone 2AX in IL-17RA deficient mice, indicating that the DNA damage response is highly activated. These data suggest that IL-17RA signaling activates a protective pathway to prevent excessive inflammation which otherwise can lead to increased oxidative stress, DNA damage, and drive gastric carcinogenesis after *H. pylori* infection.

## Introduction

*Helicobacter pylori* is the most common chronic bacterial infection world-wide and the primary risk factor for the development of gastric cancer.^1,2^ Prevalence of *H. pylori* infection differs considerably by geographical location and age, as does the incidence of gastric cancer.^3,4^ A recent meta-analysis found that approximately 79% of non-cardia gastric cancer (NCGA) and 62% of cardia gastric cancer in Asia, and 87% of NCGA in Europe and North America could be attributable to *H. pylori* infection.^5^ While not all *H. pylori-*infected persons will develop significant pathology, many develop symptomatic gastritis, some develop peptic ulcer disease, and a smaller, but significant, percentage develop gastric cancer.^6,7^ Gastric cancer is the 5^th^ most common cancer worldwide with about 1 million people diagnosed in 2022.^8^ It is the most common cancer diagnosed in eight countries and ranks 5^th^ in terms of mortality worldwide.^8^ The United States’ National Cancer Institute’s Surveillance, Epidemiology and End Results Program (SEER) reports an updated 5-year survival rate of 36.4% for gastric cancer based on data from 2014–2020.^8,9^

*H. pylori* virulence factors, environmental factors (i.e. diet), and the host’s immune response all contribute to worsened disease pathologies (reviewed in^10^). The gastritis associated with *H. pylori* infection reflects the recruitment and activation of several subsets of immune cells.^11^ In both human *H. pylori* infections and experimental murine models, a predominant pro-inflammatory mixed CD4^+^ T helper cell response is typically observed.^12,13^ While CD4^+^ T cells are critical for controlling proliferation of bacteria through the activation of innate immune cells and epithelial cells, these cells also contribute to *chronic* inflammation.^14^ Historically, IFNγ producing Th1 cells were believed to be the major driver of the gastric immunopathology associated with *H. pylori* infection. IFNγ can induce several chemokines which recruit immune cells (Cxcl9, 10 and 11) and contribute to gastritis.^15,16^ Further, IFNγ can also activate reactive oxygen species (ROS) production especially in the face of a pathogen. Moreover, IFNγ has been found to induce cell death and/or cellular senescence via ROS and DNA damage.^10^ Therefore, the role of IFNγ in carcinogenesis and tumor biology is not unambiguous since it has both pro-cancer and anti-tumor roles. About 20 years ago, Th17 cells were identified as an additional contributor to *H. pylori-*induced inflammation in the gastric mucosa (seminal observations made in^17–20^). We previously identified the IL-17RA as having a dichotomous role during *H. pylori* infection.^21–23^ IL-17RA is expressed on epithelial cells, stromal cells, fibroblasts, and lymphocytes.^24,25^ IL-17RA/IL-17Receptor C (IL-17RC) multimeric receptors are required for IL-17A, IL-17F and IL-17A/F signaling. *Il17ra*^*-/-*^ mice exhibit decreased neutrophil (PMNs) recruitment in response to acute bacterial and fungal pathogens.^21,26–29^ As a result, *Il17ra*^*-/-*^ mice are unable to control many pathogens. In the established model of chronic *H. pylori* infection in C57Bl/6 mice (which did not lead to cancer development), IL-17RA deficiency results in reduced neutrophil recruitment and elevated bacterial burden.^21^ Paradoxically, the chronic inflammatory response is exacerbated, suggesting a potential regulatory role for IL-17 signaling in preventing hyperactivation of the adaptive immune response.^21,22^ IL-17RA signaling may protect against a prolonged pathogenic T cell response and thus, IL-17RA may prevent chronic activation of damaging inflammatory pathways. Moreover, the Human Protein Atlas Project indicates that high expression of IL-17RA is a favorable prognostic marker for survival in gastric cancer patients.^30–32^ There is also evidence that several polymorphisms in the *IL17A* gene (rs2275913G>A OR = 2.21, 95% CI = 1.29-3.79)^33^ and the *Il17F* gene (rs763780C>T OR = 1.35, 95% CI = 1.16–1.58)^34^ influence susceptibility to gastric cancer.^35,36^

These data suggest that IL-17 receptor signaling, specifically mediated through IL-17RA, contributes to an immune modulation/protection during *H. pylori* infection and carcinogenesis. How to interpret the changes in expression of IL-17 and other Th17 associated markers in humans is not clear. Expressional analysis of Th17-associated markers during *H. pylori*-infection and gastric cancer (GC) demonstrated a strong positive correlation between the transcription factor associated with Th17 responses, *Rorγt*, IL-17A and IL-21 in both *H. pylori*-infected tissues and cancer tissues.^37^ The authors posit that this correlation provides evidence supporting the notion that Th17 cell expansion may contribute to cancer development. Additionally, in a recent study which investigated N-methyl-N-nitrosourea (MNU) treatment and *H. pylori* infection in a mouse model of gastric cancer, IL-17A knockout mice did not develop as many gastric tumors and had a decrease in oxidative stress.^38^ Their results suggest that IL-17A promotes gastric carcinogenesis through IL-17RC/NFκB/NOX1 activation. In another study, the relationship between expression of T helper 17 (Th17)-related cytokines, IL-17 and IL-21, to angiogenesis and clinicopathological parameters was assessed. They found that mRNA expression of IL-17 in tumors was associated with the depth of the tumors, lymph-vascular invasion, and lymph node involvement.^39^ This led the authors to suggest that IL-17 is linked to tumor progression.

Numerous cytokine receptors, including IL-17 receptors, are expressed on epithelial cells, prompting investigations into how these cells respond to T cell-derived cytokines. Our own findings suggest that the major impact of IL-17 on epithelial cells is the induction of chemokines, S100 proteins, *Nox1* and *Pigr*.^32^ However, the understanding of the impact of these cytokines on barrier function, gastric stem cell populations, or epithelial cell restitution in the stomach post-*H. pylori* infection remains limited. It is known that barrier function is mediated by a number of different molecules and cells in the mucosa, including recruitment of neutrophils (and their antimicrobial products) and immunoglobulin A (IgA). We previously published that after *H. pylori* infection, *Il17ra*^*-/-*^ mice on the FVB/n background have impaired barrier integrity exhibited by reduced mucin production and high levels of IgA in the mucosa (despite reduced *Pigr* expression) compared to infected FVB/n WT mice,^22^ but how IL-17RA impacts the development of gastric cancer was not investigated. In this study, we investigated the role of IL-17RA in *H. pylori* induced gastric cancer using the *InsGAS* mouse model. This model provides the opportunity to investigate pathological consequences of *H. pylori* infection beyond gastritis. We provide evidence that IL-17RA plays a cancer suppressive role through regulating the adaptive immune response to *H. pylori*. *H. pylori* associated hyperactive chronic Th17 and Th1 immune responses are associated with early increased bacterial burden, and they may be driven by increased antigenic uptake and presentation in the tissues and by local paragastric lymph node activity. IL-17RA deficiency in the *InsGAS* mice led to increases in expression of *Cybb*, the gene encoding NAPDH oxidase 2 (Nox2), increased DNA damage, and accelerated the development of gastric cancer in response to *H. pylori*, underscoring the protective nature of IL-17 signaling in this chronic carcinogenic microenvironment.

## Materials and methods

### Ethics statement

Institutional Animal Care and Use Committee (IACUC) of Vanderbilt University Medical Center and the Research and Development Committee of the Veterans Affairs Tennessee Valley Healthcare System approved all animal procedures in this study under protocols V1800070 and V2000068. Power analyses were provided to justify sample sizes for this study including the primary outcome of the development of gastric cancer. Animal experiments were performed in accordance with AAALAC guidelines, the AVMA Guidelines on Euthanasia, NIH regulations (Guide for the Care and Use of Laboratory Animals), and the United States Animal Welfare Act. Mice were housed in an accredited research animal facility that is fully staffed with trained personnel.

### Mice

IL-17RA deficient mice, *Il17ra*^*-/-*^ mice, (*Il17ra* ${\;}^{\mathrm{em}1\;(\mathrm{hmsa})}$) were generated using CRISPR/Cas9 methodology in FVB/n mice and are available upon request.^22^ The *InsGAS* mice (FVB/N-Tg(Ins1-GAS)1Sbr/J, JAX stock #018149) are a hypergastrinemia mouse model and were a gift from Dr. Richard Peek. The original creation of the *InsGAS* mouse is described here.^40^ These mice were crossed to generate the *InsGAS*^*tg/tg*^*Il17ra*^*-/-*^ mouse used in this study. Dual zygosity of *InsGAS* breeders was determined using Envigo services (Indianapolis, IN). The mice were *Helicobacter*-free prior to infection. Feces from sentinel mice housed in the same rooms consistently tested negative for pinworms, mouse parvovirus, and several other murine pathogens. Mice were transferred to an ABSL2 facility prior to infections. Cages of mice are randomized into infected v. uninfected groups. Mice were euthanized at predetermined time points post-infection (including uninfected mice) and tissues were collected for analysis. Male and female mice were used for some analyses where indicated. Male mice were used more frequently due to efficiency of *H. pylori* colonization. The order of treatments (e.g. infections, antibody treatments) or cage location are not considered confounding variables in our chronic experimental model.

### H. pylori *strain and mouse inoculation*

*H. pylori* PMSS1 strain (a Cag type IV secretion system positive strain) was grown from a freezer stock on 5% sheep blood Tryptic Soy Agar (TSA) plates. Cultures were passaged every 48 hours until a sufficient quantity was obtained to start liquid cultures. Two plates were used to inoculate each flask containing 50 mL of Brucella Broth with 10% Fetal Bovine Serum (FBS) and 10 µg/mL vancomycin. Liquid cultures were grown for approximately 18 hours at 160 rpm on a Maxq2000 orbital shaker (ThermoFisher Scientific, Waltham, MA) at 37°C in a BD GasPak™anaerobic chamber with EZ sachets per manufacturer’s recommendations (Becton Dickinson, Franklin Lakes, NJ). Concentration of liquid cultures was quantified via OD_600_ on BioTek ELx808 plate reader (BioTek, Winooski, VT) utilizing Gen5 3.10 software (BioTek).

At 8–10 weeks of age, if randomized to the infection group, mice were orogastrically gavaged with an inoculum of *H. pylori* PMSS1 strain at 1 × 10^9^ CFU in 0.5 mL of Brucella Broth; two doses were administered approximately 48 hours apart. Doses were verified by plating serial dilutions of the inoculums.

### Mouse tissue and sample collections

At the experimental endpoint, mice were sacrificed, and tissues were harvested for several assays. The stomach was removed, opened along the lesser curvature, and rinsed in 1 mL of PBS to derive a gastric wash sample. The stomach was sectioned into 3 or 4 longitudinal sections from the squamocolumnar junction to the duodenum, each section containing the entire length of both glandular compartments (antrum and corpus). One of these sections was used for histological analysis, one for colonization measurements, and one for gene expression analyses. In experiments where flow cytometry was performed the ½ of the stomach was used for flow cytometry while ¼ was used for colonization measurements and ¼ for either histological analysis or for gene expression. The number of replicates for experiments is indicated in the figure legends. Most experiments were performed on three different occasions.

### Bacterial colonization measurements

Approximately ¼ of the stomach was utilized to determine the level of *H. pylori* colonization. The tissue section was placed into a pre-weighed, chilled tube containing Brucella Broth with 10% FBS and five (5) stainless steel balls, and the weight of the tissue was determined. The tissue was homogenized in a FisherBrand Bead Mill 24 (ThermoFisher Scientific). The homogenate was then diluted with 10% FBS in Brucella Broth to create 10^2^, 10^3^, and 10^4^ dilutions. Diluted homogenate was plated on TSA plates containing 5% sheep blood (Hemostat Laboratories, Dixon, CA) nalidixic acid (10 µg/mL), vancomycin (50 µg/mL), amphotericin (2 µg/mL), and bacitracin (100 µ g/mL). After 6 days in an airtight container with BD GasPak™ EZ Sachets (Becton Dickinson) at 37° and 5% CO_2_, colony forming units (CFU) were counted and CFU per gram of tissue was calculated to normalize each sample to the weight of the initial gastric section. Log transformation of CFU/g was calculated, and an unpaired t-test was performed to determine significance. Our limit of detection is 4 log CFU/gram of stomach tissue.

### Histological analysis

The middle section of the harvested stomach tissue was first placed onto Whatman paper, then transferred into a histo-cassette with a foam pad. Tissue sections were fixed in 10% Neutral Buffered Formalin (NBF) for a minimum of 24 hours before processing. Five-micron thick sections were stained with hematoxylin and eosin (H&E). A single pathologist who is blinded to experimental conditions scored indices of inflammation and assessed the presence of dysplasia and cancer. Several variables were graded on a 0 to 3 scale (0, none; 1, mild; 2, moderate; 3, severe) in the gastric antrum and corpus: acute inflammation (polymorphonuclear cell infiltration) and chronic inflammation (mononuclear cell infiltration); thus, a maximum inflammation score of 12 was possible for each animal. Loss of parietal cells and loss of chief cells were scored separately as follows: 0 (no loss of cells), 1 (>0 up to 30% cell loss), 2 (>30 to 60%), and 3 (>60% loss of cell type). Corpus foveolar hyperplasia, defined as elongation of foveolae, was scored in the corpus in a 0–3 scale (absent, mild, moderate, or severe). Lymphoid follicles were defined as accumulations of lymphocytes with a germinal center. Lymphoid aggregates were defined as accumulations of lymphocytes (and other mononuclear leukocytes) without a germinal center. The combined number of lymphoid follicles and aggregates observed in H&E sections was obtained (one section per animal spanning the entire length of the glandular stomach).

As previously described,^22^ low-grade dysplasia was defined as irregular, angulated, and occasionally cystically dilated glands with enlarged overlapping hyperchromatic nuclei. Carcinoma was defined as irregular, angulated, cystically dilated glands with occasional cribriform architecture, with invasion to the lamina propria (intramucosal), to the submucosa or muscularis propria.

### Immunohistochemical staining

Five-micron thick sections (formalin-fixed, paraffin-embedded gastric specimens) were deparaffinized by routine methods. Antigen retrieval, quench, and antibody incubations were performed as previously described.^22^ In short, for anti-Muc5ac (Cat no. 36623, Cell Signaling Technology, Danvers, MA), a 1:400 dilution was used, for pH2a× (Cat no. NB100–2280, Novus Biologicals, Centennial, CO) a 1:400 dilution was used, and for anti-Ki67 (Cat no. 12202, Cell Signaling Technology), a 1:600 dilution was used. Antibody localization was performed using an HRP-labeled polymer (Cat no. K4003, Dako, Glostrup, Denmark). Staining was visualized by 5-minute incubation with chromogen diaminobenzidine (DAB+). Ki67 and pH2ax staining were quantified by counting epithelial cells with positive nuclear staining in the 5 high-power fields (400×) with the highest counts in well-oriented areas of corpus in eah animal. Because the proliferative and neoplastic changes were mainly observed in the corpus, we did not assess the antrum. Average counts per animal were tested for significance with a Welch’s t-test.

### RNA isolation and qRTPCR

RNA was isolated from stomach and paragastric lymph nodes from *InsGAS*^*tg/tg*^ and *InsGAS*^*tg/tg*^*Il17ra*^*-/-*^ mice. The stomach RNA is extracted from a longitudinal section of gastric tissue approximately ¼ to ⅓ of the total tissue, while the whole paragastric lymph node is used. Paragastric lymphnodes are manually digested in a 12 well plate with a 3 mL syringe plunger and 1 mL of TRIzol Reagent (Invitrogen, Waltham, MA). The stomachs are mechanically digested with gentleMACs Dissociator m-tubes (Miltenyi Biotec, Begisch Gladbach, Germany) and 1 mL of TRIzol. RNA was extracted according to manufacturer’s recommendations except for an additional chloroform extraction to aid in purity. At the end of the protocol, RNA clean-up was performed with the RNeasy Mini kit protocol from Qiagen (Qiagen, Hilden, Germany), and cDNA was generated using the High-Capacity cDNA reverse transcription kit from ThermoFisher Scientific. cDNA was used for real-time qRTPCR assays using Taqman Fast advanced master mix (Cat no. 4444557, ThermoFisher Scientific) and genetic probes (ThermoFisher Scientific, defined below), and a QuantStudio 6flex PCR machine (Applied Biosystems, Waltham, MA) per manufacturer’s recommendations.

Relative Units were calculated to measure relative gene abundance. Relative Units are quantified by comparing tissues from uninfected and *H. pylori* infected tissues and utilizing glyceraldehyde-3-phosphate (*Gapdh)* as an endogenous control. Relative Units are calculated by subtracting the cycle threshold (CT) of the gene of interest from the CT of *Gapdh* which yields the ΔCT. The ΔΔCT is calculated by subtracting the ΔCT of the uninfected sample from the ΔCT from an experimental sample for each gene; then 2^−ΔΔCT^ yields Relative Units. We utilized this methodology to measure genetic production for the following primer probe sets from ThermoFisher Scientific: *Il17a* (Mm00439619_m1), *Il21* (Mm00517640_m1), *Ifng* (Mm99999071_m1), *Defb14* (Mm00806979_m1), *s100a8* (Mm00496696_g1), *Pigr* (Mm00465049_m1), *Nox1* (Mm00549170_m1), *Muc5ac* (Mm01276718_m1), and *Muc6* (Mm00725165_m1), *Nox4* (Mm00627696_m1), and *Cybb* (Mm01287743_m1).

### *IgA ELISA and* H. pylori-*specific IgA ELISA*

Gastric washes were collected at the time of tissue harvest as denoted above. Gastric wash was processed to remove large debris/food by spinning at 13,000 ×g for 10 minutes at 4°C and collecting the supernatant. The concentration of IgA in the gastric wash was determined using diluted gastric wash samples (1:5, 1:25, or 1:125 in 1× assay buffer) with the ThermoFisher IgA ELISA kit as directed by manufacturer (Cat no. 88 -50450-22). The protocol was modified slightly to allow for the highest standard to be at 50 ng/mL. IgA was quantified on the BioTek ELx808 plate reader (BioTek), and standards were run in duplicate. 4- parameter logistic regression was applied to fit the unknowns to the standard curve.

For the *H. pylori-*specific ELISA, ELISA plates were coated with 10 µg/mL *H. pylori* lysate (PMSS1 strain) overnight at 4°C. Gastric wash samples were diluted 1:5, 1:25, or 1:125 in 1× PBS. Following an overnight incubation at 4°C and wash with Invitrogen ELISA wash buffer (Cat no. 00-0400-59), a goat anti-mouse IgA antibody-HRP secondary (Cat no. 1040–05, Southern BioTech, Birmingham, Alabama) was diluted 1:6000 in assay buffer and added. Signal was developed with 1X Invitrogen TMB substrate (Cat no. 00-4201-56) followed by 1 M H_2_SO_4_ stop solution. Absorbance was read at 450 nm. The data are reported as the fold above the absorbance reading of gastric wash from uninfected mice (a mixed pool of 3 uninfected samples).

### ROSALIND® Nanostring gene expression methods

Data was analyzed by ROSALIND® (https://rosalind.bio/.), with a HyperScale architecture developed by ROSALIND, Inc. (San Diego, CA). Violin plots were generated as part of the QC step. Normalization, fold changes, and p-values were calculated using criteria provided by Nanostring. ROSALIND® follows the nCounter® Advanced Analysis protocol of dividing counts within a lane by the geometric mean of the normalizer probes from the same lane. Housekeeping probes to be used for normalization are selected based on the geNorm algorithm as implemented in the NormqPCR R library.^41^ Fold changes and p-Values are calculated using the fast method as described in the nCounter® Advanced Analysis 2.0 User Manual. P-value adjustment is performed using the Benjamini-Hochberg method of estimating false discovery rates (FDR). Several database sources were referenced for enrichment analysis, including Interpro4, NCBI5, MSigDB6,7, REACTOME8, and WikiPathways9. Enrichment was calculated relative to a set of background genes relevant for the experiment.

### Tissue digestion and flow cytometry

The tissue digestion protocol was adapted from Nayar et al. 2017. Tissues of interest, specifically stomach and paragastric lymph nodes, were dissected and placed in RPMI 1640 without L-glutamine and with 2% FBS on ice for the duration of the harvest. Afterwards, tissues were digested in Collagenase P Buffer containing RPMI 1640 without L-glutamine, with 2% FBS, 0.8 mg/ml collagenase dispase (Cat no. 11097113001), 0.2 mg/ml Collagenase P (Cat no. 11213857001), and 0.1 mg/ml DNAse I (Cat no. 10104159001, Sigma Aldrich, St. Louis, MO) in a 37°C water bath for 1 hour. Next, enzymes are neutralized 1:1 with media, washed with PBS, filtered through a 70 µm cell strainer, and aliquoted into experimental tubes. Single stain controls are made with pooled cells from all samples. Experimental samples are first stained with Viability Dye (Supplemental Table 1) for 30 minutes at 4°C before being washed, centrifuged at 4°C 433 ×g for 5 minutes and resuspended in Fluorescence-Activated Cell sorting (FACs) buffer with 20% mouse serum (Cat no. 10410, Invitrogen) for staining. Antibodies used can be seen in Supplemental Table 1. Staining was performed at 4°C for 30 minutes before being centrifuged and resuspended in 300uL of FACs buffer plus 50 µL CountBright™ Plus Absolute Counting Beads (Cat. no C36995 Invitrogen) for acquisition on a Cytek Aurora 4-laser and analysis on SpectroFlo (Cytek, Fremont, CA). The gating strategy can be visualized in Supplemental Figure 6.

### IL-17a neutralization experiment

Mice were divided into four groups; uninfected and then treated with either anti-IL17A or an IgG control antibody, or infected and then treated with either anti-IL-17A or IgG control (Supplemental Figure 4A). The mice received intraperitoneal (i.p.) injections of 100ug/mouse of anti-IL17A (Clone 17F3; Cat no. BE0173 BioXcell, Lebanon, NH) or 100ug/mouse of an IgG control antibody (clone MOPC-21; Cat no. BE0083 BioXcell). The doses were given every 3 days 6 weeks post-infection for 30 days. Mice were sacrificed 14 days after the last dose (3 months post-infection).

### Statistics

Quantified data was typically graphed and analyzed with GraphPad Prism (GraphPad, San Diego, CA). CFU/g values were log transformed prior to statistical testing. When two groups were compared, an unpaired T-test was run to determine significance, but if three or more groups were run, a two-way ANOVA was used with a correction for multiple comparisons. Other statistical analyses, i.e. Fishers Exact Test, used are mentioned in figure legends as appropriate.

## Results

### *IL-17RA-deficiency leads to increased inflammation and bacterial burden after* H. pylori *infection*

**Figure 1.**
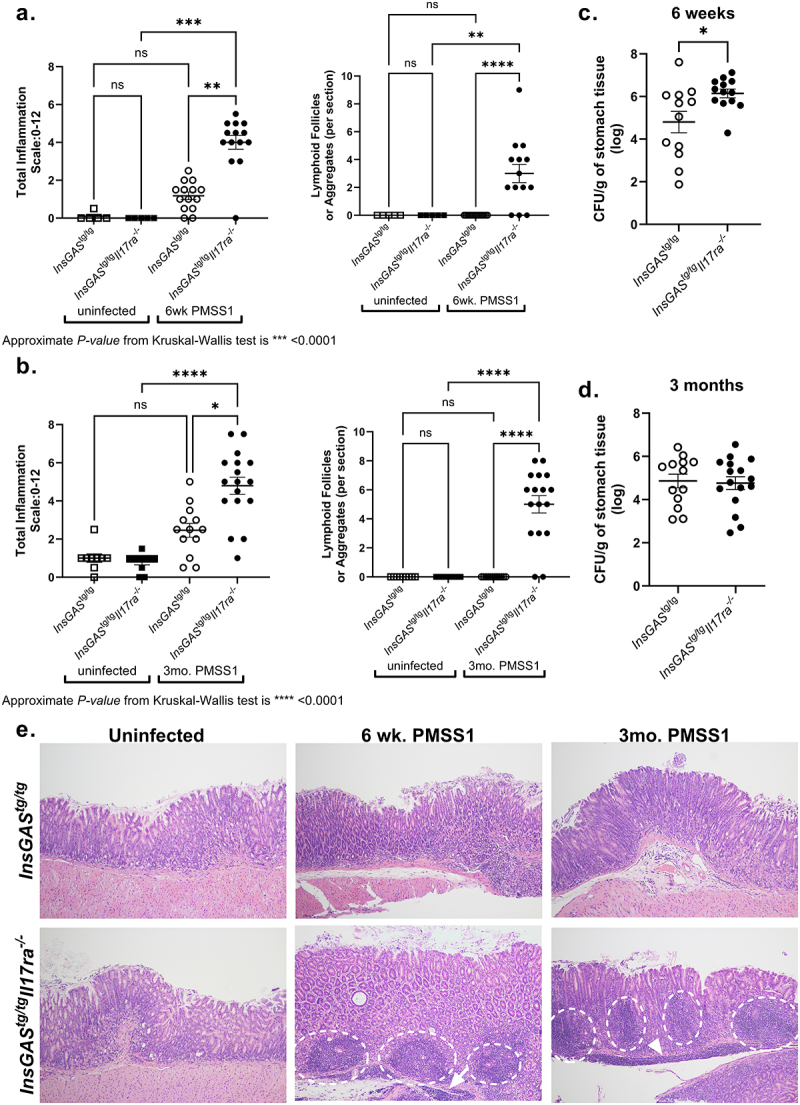
A deficiency in IL-17RA leads to increased *H.* *pylori* inflammation, lymphoid follicles, and *H.* *pylori* colonization in *InsGAS* mice. Total inflammation (combining chronic and acute inflammation) of gastric tissue and counts of lymphoid follicles and aggregates from the antrum and corpus were scored and combined to get the total per section of gastric tissue from mice infected for 6 weeks (A) and for 3 months (B) and their age-matched controls. See materials and method for scoring system. Significance was determined using a Kruskal-Wallis test. *p ≤ 0.05, **p ≤ 0.01, ****p ≤ 0.0001. Levels of *H. pylori* colonization were determined in the mice at 6 weeks (C) and 3 months (D) post-infection by plating serial dilutions of stomach homogenate. Statistical significance was determined by a t test performed on log transformed CFU/g values. These data are representative of 2 experiments at each time point. E. Representative histological images of uninfected controls compared to mice at 6 weeks and 3 months post-infection (100× magnification). Lymphoid follicles are circled; bands of lymphocytic infiltrates are indicated by arrows.

A fundamental challenge of researching the mechanisms driving gastric cancer has been the lack of an animal model that affords the ability to control/change the immune response. The insulin-gastrin *(InsGAS FVB/N-Tg(Ins1-GAS)1Sbr/J*) hypergastrinemia mouse model is an excellent model of carcinogenesis^42–44^; in response to *H. pylori* infection, these mice develop metaplasia, dysplasia, and carcinoma (originally reported^45,46^). The FVB/n background of *InsGAS* mice, however, poses an experimental barrier when investigating cytokine pathways, as many cytokine-deficient mice are on the C57Bl/6 (B6) background. To overcome this limitation, we created an *Il17ra*^−/−^ in the FVB/n background using Crispr/Cas technology,^22^ and then the *InsGAS* transgene was introgressed into the *Il17ra*^*-/-*^ mice to investigate the contribution of the IL-17RA to the development of GC in a murine model. In the absence of infection, neither the *InsGAS*^*tg/tg*^ mice nor *InsGAS*^*tg/tg*^*Il17ra*^*-/-*^ mice develop inflammation over the course of 6–8 months. (Figure 1).

**Figure 2.**
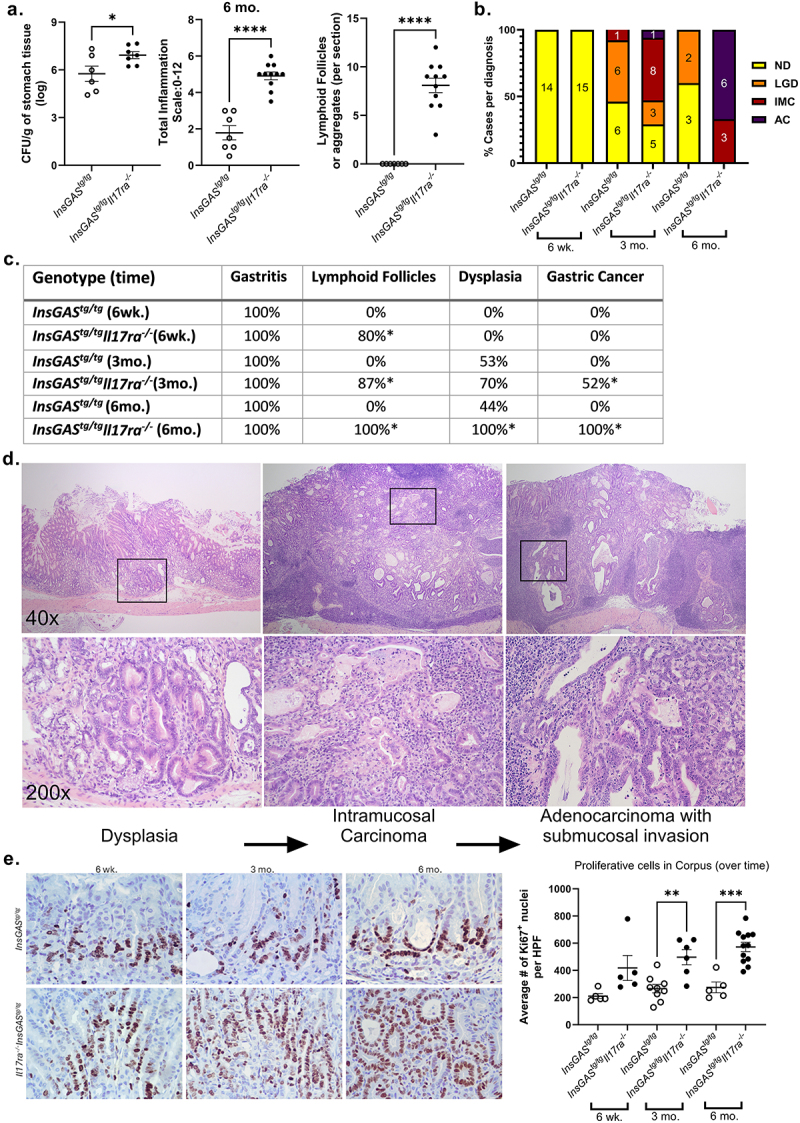
IL-17RA deficiency leads to increased cellular proliferation and development of gastric cancer in chronically infected mice. A. At the 6 mo. post-infection time point, colonization levels, total inflammation scores and number of lymphoid follicles per section are quantified. B. Frequency of adverse pathological findings in gastric tissue defined by pathologist at 3 defined timepoints post-infection of PMSS1 *H. pylori*. the numbers of cases per diagnosis are represented within each bar on the graph; diagnoses are defined as: no dysplasia (ND, gastritis only), low grade dysplasia (LGD), intramucosal carcinoma (IMC), and adenocarcinoma with invasion to the submucosa (AC). C. The table also demonstrates frequency of pathological outcomes including lymphoid follicles, dysplasia and gastric cancer. *p < 0.05, Fisher’s exact test was used to test significance of these pathological findings comparing genotypes at each time point. D. Representative H&E-stained gastric sections were imaged and are displayed at 40× (top) and 200× (bottom). Pathological outcomes of dysplasia, intramucosal carcinoma, and invasive adenocarcinoma occur in *InsGAS*^*tg/tg*^*IL-17ra*^*-/-*^ at 6-month post *H. pylori* infection and are represented from left to right. E. Ki67 staining and quantification within gastric tissues of *H. pylori* infected mice at 3 defined time points. Five high power fields (HPF) were counted per case. The average number of counts per case was graphed by genotype and time point (the average from each case is represented by the data point, mean is indicated, error bars represent ± SEM).

To model gastric carcinogenesis, mice were infected with the PMSS1 strain (Cag T4SS^+^ strain) of *H. pylori*, and the course of infection was followed for up to 6 months. By 6 weeks post-infection, *H. pylori* infection induced gastritis in both *InsGAS*^*tg/tg*^ and *InsGAS*^*tg/tg*^*Il17ra*^*-/-*^ mice. The inflammation scores were significantly higher in *H. pylori* infected *InsGAS*^*tg/tg*^*Il17ra*^*-/-*^ mice compared to uninfected mice or *H. pylori* infected *InsGAS*^*tg/tg*^ mice. Moreover, by 6 weeks post-infection, lymphoid follicles were commonly observed in the infected *InsGAS*^*tg/tg*^*Il17ra*^*-/-*^ mice, but not in uninfected mice or infected *InsGAS*^*tg/tg*^ mice (Figure 1(a), Table in Figure 2(c)). At this same time point, there was a significant increase in *H. pylori* burden in the stomachs of the *InsGAS*^*tg/tg*^*Il17ra*^*-/-*^ mice compared to *InsGAS*^*tg/tg*^ mice (Figure 1(a)). At 3 months post-infection, the differences were very pronounced. *InsGAS*^*tg/tg*^*Il17ra*^*-/-*^ mice exhibited significantly higher levels of inflammation associated with lymphoid follicle development in the gastric tissue compared to infected *InsGAS*^*tg/tg*^ mice (Figure 1(b), Table in Figure 2(c)). We did not observe a significant difference in bacterial burden at 3 months post-infection (Figure 1(d)). Representative H&E are presented to illustrate the inflammation and the lymphoid follicle development in the gastric tissue (Figure 1(e)). At 6 months post-infection, while there is no difference in colonization levels, chronic inflammation remained significantly higher in the *InsGAS*^*tg/tg*^*Il17ra*^*-/-*^ mice, and these mice had significantly more lymphoid follicles in their gastric mucosa compared to *InsGAS* mice (Figure 2(a,c)).

It was previously observed that *H. pylori* associated gastric cancer in *InsGAS* mice was impacted by the sex of the mice.^44,47–49^ To investigate sex as a biological variable in the hyperinflammatory response that develops in *InsGAS*^*tg/tg*^*Il17ra*^*-/-*^ mice, we compared the response to *H. pylori* infection in male and female *InsGAS*^*tg/tg*^*Il17ra*^*-/-*^ mice at 3 months post-infection. The data indicate that female mice were not successfully colonized as frequently as male mice (Supplemental Figure 1). However, when successfully colonized, both female and male mice developed comparable, strong inflammatory responses. Namely, both male and female *InsGAS*^*tg/tg*^*Il17ra*^*-/*-^ mice often developed lymphoid follicles in response to *H. pylori* infection. Due to the lower infection rate of female mice, it was difficult to perform statistical analysis; therefore, in subsequent studies, we used *H. pylori* infected male mice to investigate the role of IL-17RA in cancer.

### IL-17RA plays a role in suppression of gastric cancer pathologies including gastric cancer development

To investigate carcinogenesis in this model, gastric tissues were scored for several pathological outcomes including corpus atrophy (loss of parietal and chief cells), corpus foveolar hyperplasia, dysplasia, and gastric cancer (with either intramucosal or submucosal invasion) at 6 weeks, 3 months, and 6 months post-infection (Figure 2(b) and Supplemental Figure S2). By 6 weeks post- infection, the absence of IL-17 signaling is associated with an increase in parietal cell loss, which is maintained throughout chronic infection and disease. The loss of chief cells progressed with *H. pylori* infection independently of IL-17RA, especially between 6 weeks and 3 months post-infection (Supplemental Figure S2). While there was a small, but significant increase in the presence of corpus foveolar hyperplasia at 6 weeks post-infection in the *InsGAS*^*tg/tg*^*Il17ra*^*-/-*^ mice compared to *InsGAS*^*tg/tg*^ mice, there was no difference at later time points. One of the most striking findings in this study is that *H. pylori-*infected *InsGAS*^*tg/tg*^*Il17ra*^*-/-*^ mice developed dysplasia and gastric cancer more frequently by 3 months and 6 months post-infection compared to infected *InsGAS*^*tg/tg*^ mice (Figure 2B,C). In fact, about ½ of the IL-17RA deficient mice developed gastric cancer by 3 months post-infection and all of the *H. pylori -*infected *InsGAS*^*tg/tg*^*Il17ra*^*-/-*^ mice developed gastric cancer by 6 months post-infection- a time point when *H. pylori* infected *InsGAS* mice did not develop pathology beyond low grade dysplasia. Representative micrographs of low-grade dysplasia, intramucosal carcinoma, and adenocarcinoma with submucosal invasion are presented in Figure 2(d). The gastric pathology observed in the *InsGAS*^*tg/tg*^*Il17ra*^*-/-*^ mice is quite striking; therefore, we present both high and low magnification micrographs of the H&Es.

Ki67 is expressed in the nuclei of actively proliferating cells and thus, Ki67 staining serves as effective marker for cell proliferation in many tumors. Cellular proliferation is believed to increase the rate of tumorigenesis. Gastric tissues were stained for Ki67 at 3 time points post-infection in both *InsGAS* mice and *InsGASIl17ra*^*-/-*^ mice (Figure 2E). In the infected *InsGASIl17ra*^*-/-*^ mice, a significant increase in Ki67 epithelial cell staining was observed compared to *InsGAS* mice at 3 mo. and 6 mo. post-infection.

While previous studies investigating how IL-17RA deficiency (*Il17ra*^*-/-*^ mice) impacts *H. pylori-*induced pathology demonstrated increased inflammation and an impact on barrier function in the absence of IL-17RA, the most chronic time point investigated in those studies was 3 months post- infection.^22^ To test the hypothesis that a longer time course would lead to dysplasia or cancer, *Il17ra*^*-/-*^ and wild type mice (FVB/n) were infected with PMSS1 for 6 months. At this time point, there was no significant difference in colonization and an increase in total inflammation in *Il17ra*^*-/-*^ mice (Supplemental Figure 3A). Further, consistent with observations in *InsGAS*^*tg*^ mice, *Il17ra*^*-/-*^ mice developed more severe disease than wild type mice. All *H. pylori* infected *Il17ra*^*-/-*^ mice developed dysplasia or cancer and the *H. pylori* infected wild type mice only developed gastritis (Supplemental Figure 3(b)). Representative H&Es are presented with normal mucosa (WT), mild gastritis (WT) and low-grade dysplasia (*Il17ra*^*-/-*^) (Supplemental Figure 3(c)). Together these data suggest that IL-17RA limits pathology and the development of dysplasia and gastric cancer.

**Figure 3.**
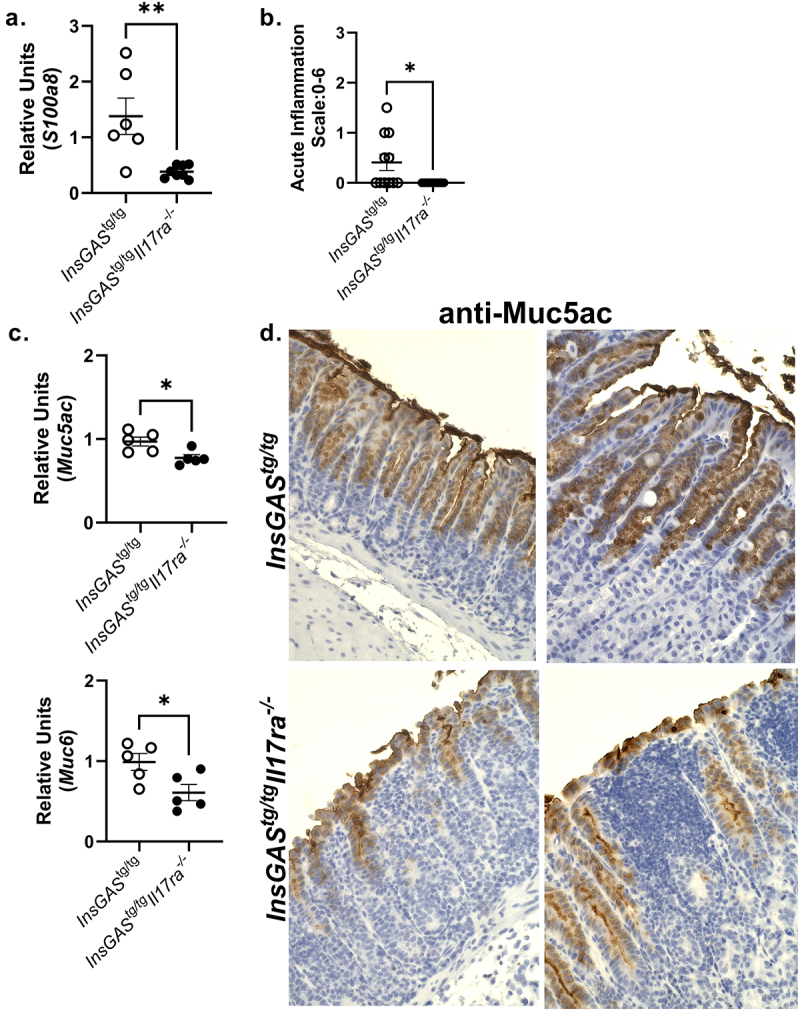
Molecules with antimicrobial function are impacted in IL-17RA deficient mice during *H. pylori* infection. A. Realtime rtPCR was performed on gastric tissue at 6 weeks post-infection for *S100a8*, a component of calprotectin. Expression is presented relative to the housekeeping gene *Gapdh* and to uninfected *InsGAS* mice (calibrator sample). B. PMN infiltration was scored as acute inflammation on a scale of 0-6 at this same time point. C. Realtime rtPCR was performed on gastric tissue at 6 weeks post-infection for *Muc5ac* and *Muc6*. Expression is presented relative to the housekeeping gene *Gapdh* and to uninfected *InsGAS* mice (calibrator sample). D. Immunohistochemical stainings for Muc5ac were performed on tissues at 3 mo. post-infection. Representative images (200×) are from 2 different mice per genotype. Error bars represent ± SEM; *p ≤ 0.05, **p ≤ 0.01.

### *Neutralization of IL-17A does not impact inflammatory response or carcinogenesis in the InsGAS mice infected with* H. pylori

The role of IL-17A, one of the 3 cytokines that utilize IL-17RA to signal responses, in carcinogenesis is controversial. Previous studies suggest that IL-17A could activate angiogenesis and/or increase metastasis.^37,38,50^ With increasing interest in the use of biological immune activators during cancer treatment or immunosuppressants during chronic disease,^51,52^ we investigated whether treating *H. pylori* infected *InsGAS* mice with anti-IL-17A would impact *H. pylori* induced disease progression. The mice were divided into four groups; uninfected and then treated with either anti-IL17A or an IgG control antibody, or *H. pylori* infected and then treated with either anti-IL-17A or IgG control (Supplemental Figure S4A). *H. pylori* colonization levels did not differ between mice receiving anti-IL17A compared to those receiving IgG. Inflammation developed in the *InsGAS* mice by 3 months post-infection and the level of inflammation was not impacted by antibody treatment (Supplemental Figure S4B). These data suggest that neutralization of IL-17A alone for an acute period (6 weeks) did not impact inflammation or disease progression at 3 months post-infection.

### *Innate components of the mucosal response may be compromised during* H. pylori *infection when there is IL-17RA deficiency*

Our recent characterization of *H. pylori* infection in *Il17ra*^−/−^ mice on the FVB/n background revealed an impact on antimicrobials and mucin production.^22^ To determine if these pathways are impacted in the *InsGAS*^*tg/tg*^ mice, real time rtPCR was performed for several antimicrobial factors including *S100a8, Muc5ac*, and *Muc6*. In uninfected mice, there were no significant differences in the relative units of *Muc5ac* or *Muc6* expression (Supplemental Figure S5A). However, based on Nanostring analysis, uninfected *InsGAS*^*tg/tg*^*Il17ra*^*-/-*^ mice exhibited significantly lower transcript levels of *S100a8* compared to uninfected *InsGAS*^*tg/tg*^ mice (See Supplemental Table 2, Supplemental Figure S5B). After *H. pylori* infection, there was significantly lower expression of *S100a8, Muc5ac*, and *Muc6* transcripts in *H. pylori* infected *InsGAS*^*tg/tg*^*Il17ra*^*-/-*^ mice compared to *H. pylori* infected *InsGAS*^*tg/tg*^ mice (Figure 3(a,c)). Since S100a8 is a major component of calprotectin, a neutrophil marker, the decreased expression of *S100a8* likely signals a reduction of neutrophil recruitment in the absence of IL-17RA. To address if this was a marker of reduced neutrophil recruitment, the acute inflammation was scored. Based on the pathologist’s scoring, there were no neutrophils recruited to the gastric mucosa of *InsGAS*^*tg/tg*^*Il17ra*^*-/-*^ mice; in the absence of IL-17RA they do not develop acute inflammation (6 weeks post-infection, Figure 3(b)). To further investigate the reduced expression of *Muc5ac*, we stained gastric tissue at 3 months post-infection for the Muc5ac glycoprotein by immunohistochemistry (Figure 3(d)). There is less Muc5ac staining in the *InsGAS*^*tg/tg*^*Il17ra*^*-/-*^ mice compared to *InsGAS* mice, with evidence that the gastric glands have become disorganized as immune cell infiltrate disrupts the architecture. The gastric tissue of the *InsGAS*^*tg/tg*^*Il17ra*^*-/-*^ mice is much thicker due to both the immune cell infiltrate and potential hyperplasia in the epithelial cells. These data suggest that there are changes in tissue architecture and reduced abundance of mucins in the *InsGAS*^*tg/tg*^*Il17ra*^*-/-*^ mice compared to *InsGAS* mice.

### Gene expression profiling of gastric tissue supports role for IL-17RA in regulating antigen presentation and processing

To investigate changes in the tumor biology, microenvironment, and the immune response in the stomach tissues of *InsGAS*^*tg/tg*^ and *InsGAS*^*tg/tg*^*Il17ra*^*-/-*^ mice, gene expression profiling was performed on RNA isolated from whole gastric tissue using the Nanostring nCounter PanCancer IO 360™ Panel. This panel measured the expression of 770 genes, including 20 housekeeping genes. The analysis allowed us to identify genes which are up or down regulated in gastric tissues in the absence of IL-17RA with and without *H. pylori* infection. In uninfected mice, minimal differential gene expression was observed, with only 6 genes being up or downregulated greater than 2-fold (adj p-Value of ≤ 0.01, Supplemental Table 2, Supplemental Figure S5B).

**Figure 4.**
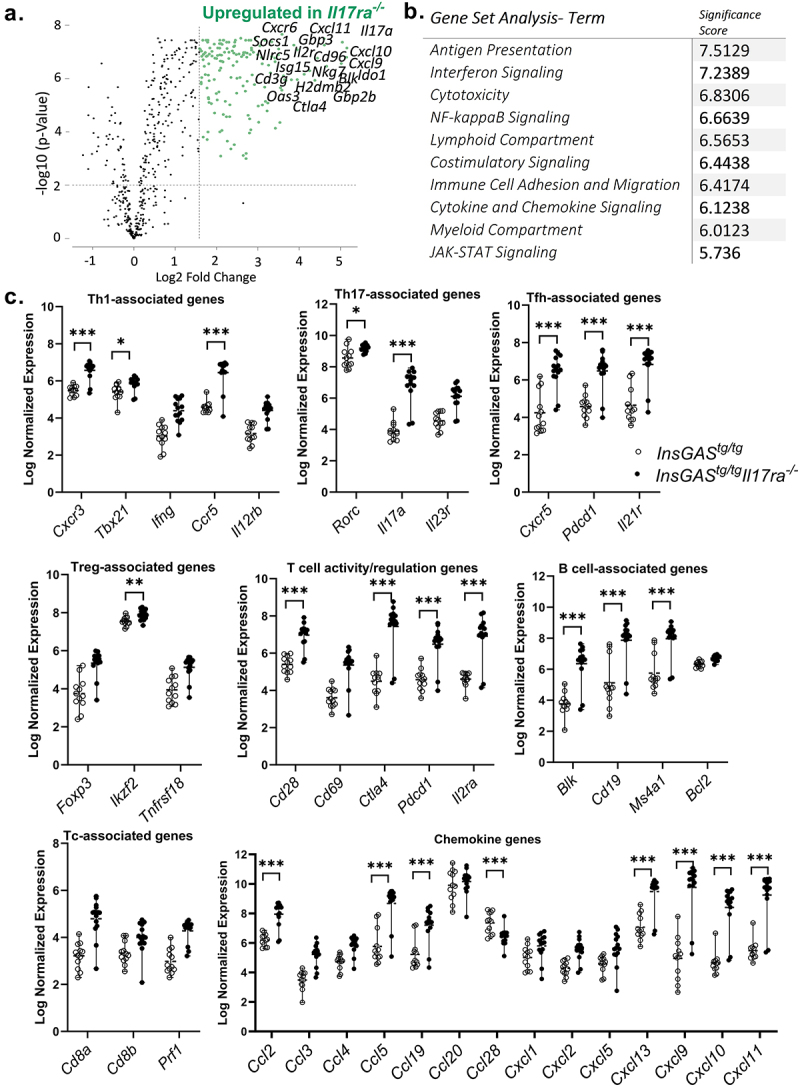
PanCancer IO 360 gene expression analysis panel data supports changes in antigen presentation pathways and lymphocyte activation. A. Volcano plot of differentially expressed genes in stomach tissue from *InsGAS*^*tg/tg*^ compared to *InsGAS*^*tg/tg*^*Il17ra*^*-/-*^ tissue (at 3 mpi, 5 mo of age). Differential gene expression was determined by analyzing data generated using the Nanostring PanCancer IO360 nCounter panel using *n* = 12-14 samples per genotype. B. Gene set annotation analysis indicates that a significant number of genes associated with antigen presentation, interferon signaling, cytotoxicity, NFkB signaling are upregulated. C. Graphical representations of normalized expression values from Nanostring data set for lymphocyte subsets (Th1, Th2, Th17, Treg, Tfh, B cells) and chemokines. Statistical differences were determined using differential gene expression analysis and * p-adj <0.05, ** p-adj <0.001 and *** p-adj <0.0001. Supplemental table 3 also contains differential gene expression data.

Analysis of transcript abundance in samples from *H. pylori* infected *InsGAS*^*tg/tg*^ and *InsGAS*^*tg/tg*^*Il17ra*^*-/-*^ mice at 3 months post-infection indicate that there are 162 genes upregulated greater than 3-fold (>1.59 log^10^ fold, with adjusted p-Value ≤0.01, Supplemental Table 3). There were no genes significantly down regulated with an adjusted p-Value ≤0.01. A volcano plot illustrates how striking this shift in gene expression is in the *H. pylori-*infected *InsGAS*^*tg/tg*^*Il17ra*^*-/-*^ mice (Figure 4(a)). Further analysis of these differentially regulated pathways indicate that genes associated with antigen presentation have the most significant upregulation as a group, according to Nanostring annotations (significance score of 7.5129), followed by genes associated with interferon signaling, cytotoxicity, and NFkB signaling (Figure 4(b)). Rosalind, as a data analysis tool, can draw from several other pathway analysis packages including BioPlanet, Panther, and Reactome. These pathway analyses suggest that T cell activation pathways and immune regulatory pathways are upregulated in the *InsGAS*^*tg/tg*^*Il17ra*^*-/-*^ mice compared to *InsGAS*^*tg/tg*^ mice (Supplemental Table 4).

Based on these pathway analyses, we inquired as to how different subsets of lymphocytes were impacted by the IL-17RA deficiency. The normalized expression of genes associated with different subsets of immune cells was examined from the same gene expression array. Many genes associated with Th1, Th17, T follicular helper (Tfh) cells are expressed at a significantly higher level in *H. pylori* infected *InsGAS*^*tg/tg*^*Il17ra*^*-/-*^ mice compared to *H. pylori* infected *InsGAS* mice. These included Th1-associated genes, *Cxcr3, Tbx21* (which encodes Tbet), and *Ccr5;* Th17-associated genes, *Rorc* (which encodes Rorgt), and *Il17a;* Tfh-associated genes *Cxcr5, Pdcd1*, and *Il21r* (Figure 4(c)). Moreover, many genes associated with T activation or T cell regulation, but not necessarily associated with specific subsets of lymphocytes, were significantly upregulated including *Cd28, Ctla4, Pdcd1* (which encodes Pd1), and *Il2ra* (which encodes CD25). There were few significant differences between Treg associated genes. No significant differences were measured in expression of *Foxp3* or *Tnfrsf18-* which encodes Gitr, but there was a significant increase in *Ikzf2* which encodes helios. CD8^+^ (cytotoxic T cell, Tc)-associated genes including *Cd8* or *Prf1* (which encodes perforin) were not significantly different between genotypes. On the other hand, B cell-associated genes including *Blk, Cd19*, and *Ms4a1* (which encodes CD20) had significantly higher expression in the *H. pylori* infected *InsGAS*^*tg/tg*^*Il17ra*^*-/-*^ mice compared to *H. pylori* infected *InsGAS* mice. Together, the pathway analysis and the differential gene expression analysis data support the notion that IL-17RA deficiency in *InsGAS* mice is associated with increased antigen uptake, processing, and presentation followed closely by activation of antigen-specific lymphocytes.

To consider how extensive inflammation in the IL-17RA deficient mice may be driven by cellular recruitment to the gastric tissues, the normalized expression of several chemokines from this Nanostring panel were graphically presented (Figure 4C). Several chemokines, which are known to be regulated by IFNγ and TNF, have significantly higher levels of normalized expression in the *H. pylori* infected *InsGAS*^*tg/tg*^*Il17ra*^*-/-*^ mice compared to *H. pylori* infected *InsGAS* mice including *Ccl2, Ccl5, Cxcl9, Cxcl10*, and *Cxcl11*. These chemokines regulate cellular recruitment through CCR5 and CXCR3, receptors which are also differentially expressed in this data set (Figure 4(c) and Supplemental Table 3). B cell recruiting chemokine genes, *Ccl19* and *Cxcl13*, are also significantly higher in the *InsGAS*^*tg/tg*^*Il17ra*^*-/-*^ mice, but *Ccl28* is significantly lower in its normalized expression. *Ccl28* has been previously reported to be upregulated by IL-17 signaling.^21^ There are several other chemokines in the data set – those with significant differential expression (at a level of a > 3-fold change (>1.59 Log fold change) and a p-Adj Value < 0.01, in Supplemental Table 3) and some that are not significantly different can be visualized in Figure 4(c).

### *IL-17RA deficiency leads to increased activation of antigen specific responses to* H. pylori

**Figure 5.**
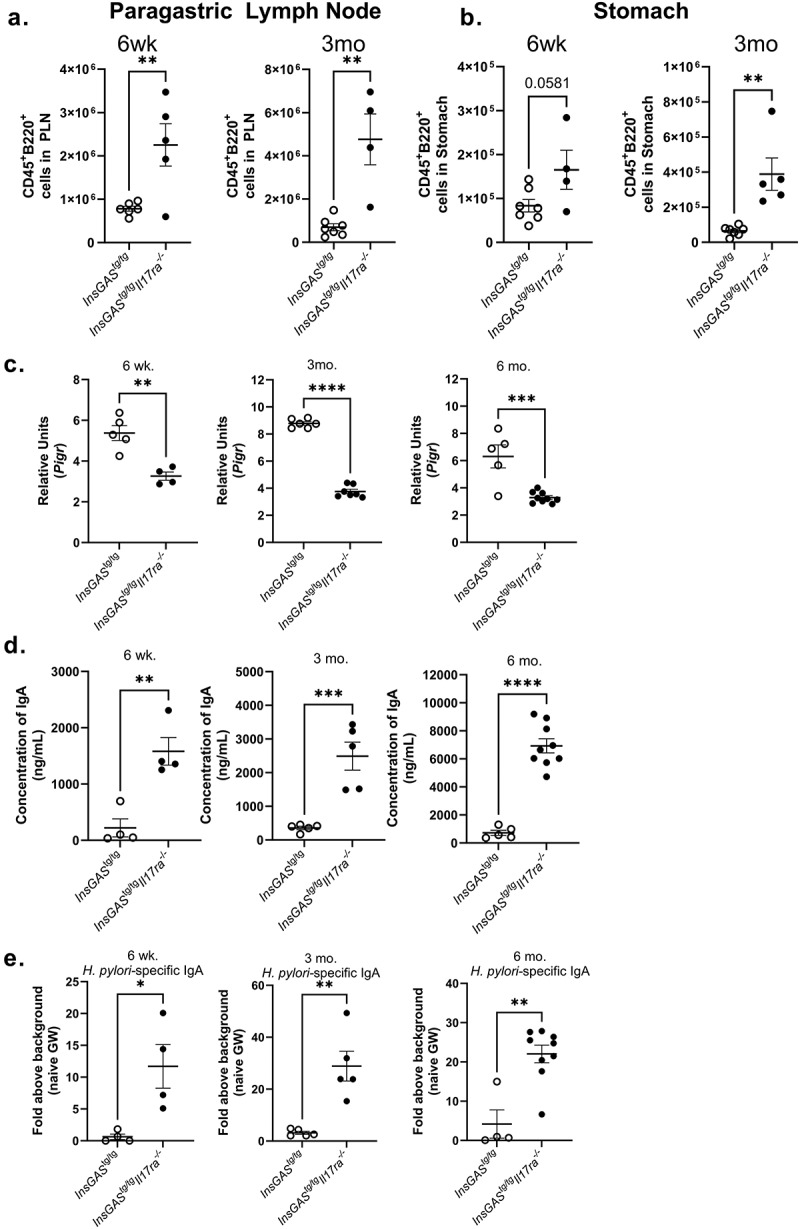
B cell responses are amplified in the IL17RA deficient mice. Paragastric lymph nodes (A) and stomach tissue (B) were digested and analyzed via flow cytometry with viability dye and counting beads for absolute cell counts of B cells (CD45^+^B220^+^) at 6-week and 3-month timepoints. The gating scheme is represented in Supplemental Figure 4. Significance was determined via an unpaired t-test. C. At three time points post-infection qRTPCR was performed to determine levels of *Pigr* expression. *Gapdh* was used as an endogenous control, and RNA pooled from uninfected mice was used as a reference sample. Relative units are calculated as described in the materials and methods section. D. Total IgA was calculated utilizing a total IgA ELISA assay providing total IgA levels in the gastric wash. E. *Hp* specific IgA levels in the gastric wash were determined and levels are reported as the fold increase above the signal from a pooled gastric wash sample from uninfected mice. Significance was determined via an unpaired t-test. Error bars represent ± SEM; *p ≤ 0.05, **p ≤ 0.01, ***p ≤ 0.001, ****p ≤ 0.0001, compared with *InsGAS* mice.

Increased inflammation scores in the gastric tissue and the differential gene expression data from the PanCancer IO 360 analysis are indicators that there is increased immune cell infiltration to the stomach. To quantify the activation of the humoral immune response and B lymphocyte infiltration to the stomach during *H. pylori* infection in this model, flow cytometry was performed on the paragastric lymph node and stomach tissue. By 6 weeks post-infection, there were significantly increased numbers of B cells (CD45^+^B220^+^) in the paragastric lymph nodes of *InsGAS*^*tg/tg*^*Il17ra*^*-/-*^ mice compared to *InsGAS*^*tg/tg*^ mice (Figure 5(a)). While there are increasing B cells in the paragastric lymph node at 6 weeks post-infection, a significant increase in B cells in both the paragastric lymph node and the stomach was evident only after 3 months post-infection (Figure 5(b)).

Gene expression of the polymeric immunoglobulin receptor (*Pigr*) is regulated by IL-17A in the intestines and in the stomach.^22,53^ To investigate if *Pigr* transcript levels were impacted in the *InsGAS*^*tg/tg*^*Il17ra*^*-/-*^ mice, real-time rtPCR was performed at 6 weeks, 3 months, and 6 months post-infection. The data indicate that relative expression of *Pigr* is significantly lower in IL-17RA deficient mice compared to *InsGAS* mice at these time points (Figure 5(c)). Interestingly, despite reduced *Pigr* expression, antibody responses are still elevated in the *H. pylori* infected *InsGAS*^*tg/tg*^*Il17ra*^*-/-*^ mice compared to infected *InsGAS*^*tg/tg*^ mice. The concentration of IgA and the relative levels of *H. pylori* specific IgA were measured in gastric wash preparations by ELISA (Figures 5(d,e)). *H. pylori* infection increases IgA levels more significantly in the *InsGAS*^*tg/tg*^*Il17ra*^*-/-*^ mice compared to the *InsGAS*^*tg/tg*^ mice at all time points investigated (Figure 5(d)). As a whole, these data indicate that that antigen specific B cell responses are significantly higher in *H. pylori* infected *InsGAS*^*tg/tg*^*Il17ra*^*-/-*^ mice compared to *InsGAS*^*tg/tg*^ mice. The increase of B cells in the gastric mucosa are likely contributing to the abundance of IgA that is found in the gastric wash. One possible explanation for these findings, considering the finding that *InsGAS*^*tg/tg*^*Il17ra*^*-/-*^ mice have lower but not ablated *Pigr* expression, is that increased antigenic load in the *InsGAS*^*tg/tg*^*Il17ra*^*-/-*^ mice combined with changes in acute inflammatory cell infiltration and expression of mucin may lead to an increase in antigen uptake and increases in B cell activation in both the lymph nodes and in lymphoid follicles.

**Figure 6.**
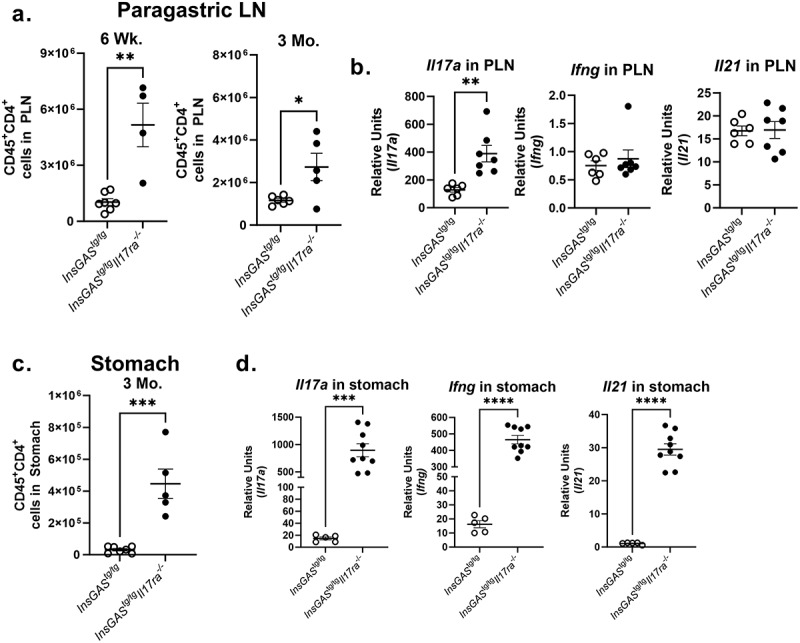
Th1 and Th17 responses are amplified as infection progresses in the absence of IL-17RA. A. Paragastric lymph nodes were digested and analyzed via flow cytometry with viability dye and counting beads for absolute cell counts of T cells (CD45^+^CD4^+^) at 6-week and 3-month timepoints. B. *Il17a, Ifng, and Il21* transcript levels were also measured in the paragastric lymph nodes (relative units was calculated using 2^−ΔΔCt^ method and uninfected PLN from *InsGAS* mice was used as the calibrator sample and *Gapdh* as the housekeeping gene). C. A standardized half section of stomach tissue was digested at 3 months post-infection for flow cytometry similar to the PLNs. The gating schemes are represented in Supplemental Figure 4. Significance was determined via an unpaired t-test. D. At 3 months post-infection, qRTPCR was performed to determine levels of *Il17a, Il21* and *Ifng* expression in gastric tissue. For all panels significance was determined via an unpaired t-test. Error bars represent ± SEM; *p ≤ 0.05, **p ≤ 0.01, ***p ≤ 0.001, ****p ≤ 0.0001, compared with *InsGAS* mice.

Considering the impact CD4^+^ T cells have on many immune processes, from anti-microbial responses to regulation of chronic inflammation, and the striking changes in T helper cell subset-associated genes (Figure 4), CD4^+^ T cell infiltration and cytokine production was explored further in the gastric tissue and paragastric lymph nodes. CD4^+^ T cell numbers were quantified in these tissues at 6 weeks and 3 months post-infection. There were significantly more CD4^+^ T cells in the paragastric lymph nodes of *InsGAS*^*tg/tg*^*Il17ra*^*-/-*^ mice compared to infected *InsGAS*^*tg/tg*^ mice at both time points (Figure 6(a)). By 3 months post-infection, there were also significantly more CD4^+^ T cells in the gastric tissue in the *InsGAS*^*tg/tg*^*Il17ra*^*-/-*^ mice compared to infected *InsGAS*^*tg/tg*^ mice (Figure 6C). The increase in CD4^+^ T cells at 3 months post-infection was accompanied by increased gene expression of *Il17a, Il21* and *Ifng* in the gastric tissues, along with increased *Il17a* in the paragastric lymph node as measured by real-time rtPCR (Figure 6(b,d)). Gene expression analysis of tissue from uninfected mice demonstrates an increase in baseline *Il17a* expression in the stomach of IL-17RA deficient mice (Supplemental Figure S5C), as previously reported in C57Bl/6 *Il17ra*^*-/-* 22^. These data suggest that IL-17 signaling is responsible for modulating the inflammatory infiltrate to the gastric mucosa in the setting of *H. pylori* infection.

### *Oxidative stress pathway is highly expressed and DNA damage signal activated in the absence of* *IL-17RA in* H. pylori *infected mice*

**Figure 7.**
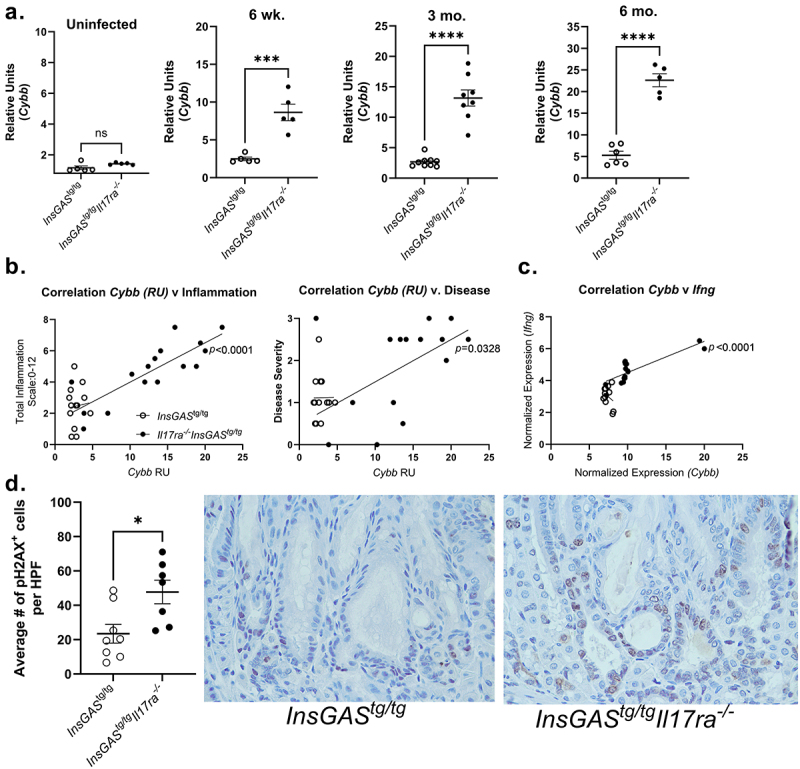
**Levels of *Cybb* and DNA damage increase in IL-17ra deficient mice after *H. pylori* infection**. A. Realtime rtPCR was performed on RNA from gastric tissue to determine relative expression of *Cybb*. An unpaired T test was performed on data at each timepoint in panel A. B. The correlations between relative expression of Cybb, inflammation scores, disease severity are graphed with data from 3 months post-infection. Student tTest; ***p < 0.001, ****p < 0.0001. C. Correlation between log normalized expression of *Cybb* and log normalized expression of *Ifng* as determined using the PanCancer IO 360 nanostring panel are graphed. Simple linear regression analyses were performed on data in panels B and C. D. pH2AX quantification, average number of positive nuclei per high power field (HPF, average of 5 per case), and representative staining in gastric tissues (magnification is 400×). Welch’s *t*-test *p < 0.05.

Chronic inflammation can drive oxidative stress and DNA damage. While reactive oxygen species contribute to host immune defense, wound healing, and cell proliferation and differentiation, aberrant levels of NOX-derived ROS may contribute to carcinogenesis. Moreover, there is published data suggesting that IL-17A promotes gastric carcinogenesis in the NMU-*H. pylori* infection model through IL-17RC/NFkB/NOX1 activation.^38^ Therefore, the expression of *Nox1, Cybb* (which encodes Nox2), and *Nox4* were measured over the 6-month time course by real-time rtPCR. There were no consistent differences in expression of *Nox1* or *Nox4* (Supplemental Figure 5(d)) at any time point after *H. pylori* infection. On the other hand, there was significantly greater expression of *Cybb* in *InsGAS* mice with IL-17RA deficiency compared to *InsGAS* mice at all three time points post *H. pylori* infection (Figure 7(a)). Increased expression significantly positively correlated with both the level of inflammation and the severity of disease in the *InsGAS*^*tg/tg*^*Il17ra*^*-/-*^ mice but did not significantly correlate with these same factors in the *InsGAS* mice without IL-17RA deficiency (Figure 7(b)). The significant increase in *Cybb* was also observed in the Nanostring analysis (Log Fold Change = 2.19, Adj p-Value <0.0001, Supplemental Table 3). Since IFN-γ is a known driver of *Cybb* expression,^54^ the correlation between Normalized Expression of these two genes was tested and there was again a significant correlation (Figure 7C). Considering data which demonstrate that Nox2 positive gastric cancers are associated with poor prognosis and correlate with pro-carcinogenic molecules such as VEGF and EGFR,^55^ the elevated *Cybb* expression in our model of rapid carcinogenesis may suggest that the increased Th1 cell activation may drive oxidative stress in this context.

Oxidative stress and specifically NOX2-derived ROS can impact cancer-promoting DNA damage.^56^ To investigate DNA damage in gastric tissues, immunohistochemistry for phosphorylated histone 2AX (pH2AX),^57,58^ a biomarker for DNA double-strand breaks, was used. There were significantly more pH2AX^+^ nuclei in gastric epithelial cells of the corpus in *H. pylori* infected *InsGAS*^*tg/tg*^*Il17ra*^*-/-*^ mice compared to *H. pylori* infected *InsGAS* mice at 3 months post-infection (Figure 7D). These data suggest that increased Nox2 (*Cybb)* may contribute to DNA damage, which is associated with more severe disease outcomes, including the development of dysplasia and cancer in IL-17RA deficient mice.

## Discussion

In this study, we demonstrate that the loss of the IL-17 receptor A is associated with earlier progression to gastric cancer in the *H. pylori* infected *InsGAS* mice. Further, the data suggests that changes at the mucosal barrier and PMN recruitment may lead to increased activation of antigen presentation pathways in the gastric tissue, as well as increased expansion of T cell and B cell responses in local lymph nodes. Increases in Th1 and Th17 cytokines in the absence of IL-17RA are associated with earlier loss of parietal cells, activation of the Nox2 gene, *Cybb*, and increased DNA damage (pH2AX). Taken together, these data suggest IL-17RA is vital for controlling chronic inflammation in the gastric mucosa, ultimately reducing architectural changes in the tissue, reducing DNA damage, and slowing the development of *H. pylori* induced gastric cancer.

The IL-17RA molecule can pair with several different IL-17 receptor subunits to provide a functional receptor. In the absence of IL-17RA, there is a loss in cytokine signaling for IL-17a, IL-17f and IL-17a/f due to the loss of IL-17RA/RC complexes. IL-17RA can also pair with IL-17RB to create the functional receptor of IL-17E (also known as IL-25).^59^ Interestingly, our prior studies examining cytokine signaling in gastric epithelial cells, gastroids, and gastric fibroblasts suggest that IL-17a is the strongest inducer of most cellular responses mediated by IL-17RA, and IL-17f does not stimulate a measurable response.^23,32^ For this reason, it is surprising that *Il17a*^*-/-*^ mice and neutralization of IL-17a does not have nearly the significant impact on *H. pylori* induced inflammation or gastric cancer development as the IL-17RA deficiency does.^23,38^ A recent publication by Kang et al. utilized *Il17a*^−/−^ mice and wild-type mice to address the role of IL-17a in gastric cancer development.^38^ They induced gastric cancer using *N-*methyl-*N*-nitrosourea (NMU) treatment in combination with *H. pylori* infection, and at nearly 1 year after treatment, they observed that *Il17a*^*-/-*^ mice had decreased oxidative stress and lower expression of gastric epithelial stem cell markers. This contrasts with our findings that IL-17RA deficiency is detrimental, and we are unable to determine why the IL-17RA receptor expression is so key for the protective phenotype with *H. pylori* infection, but it is likely due to this receptor also responding to IL-17f, having an impact when it is not ligated by a cytokine, or responding to a yet unidentified cytokine. Furthermore, their *in vitro* work with AGS (gastric epithelial) cells suggests that recombinant IL-17 drives ROS production (through *Nox1*). They do report an increase in IL-17RC with rIL17a treatment of AGS cells, and an increase in IL-17RC in human gastric cancer tissue compared to healthy tissue. Their interpretation is that IL-17a promotes carcinogenesis through regulating IL-17RC and oxidative stress, but how IL-17RA and IL-17RC complex for this mechanism is not addressed. So, while we have observed no significant changes in expression of IL-17RC or IL-17f in our studies, we are not surprised by their findings. In our previous in vitro studies, we also observed a correlation between IL-17a signaling and Nox1 expression,^23^ and *Il17ra*^*-/-*^ mice infected with *H. pylori* express reduced levels of *Nox1* compared to *H. pylori* infected wild type mice.^22^ The lower levels of *Nox1* are not associated with decreased inflammation; in fact, at 3 months post-infection, a few *Il17ra*^*-/-*^ mice develop dysplasia.^22^ Further, murine gastroids stimulated with IL-17a respond with increased *Nox1* expression.^32^ In the current study, *InsGAS*^*tg/tg*^*Il17ra*^*-/-*^ mice do not express significantly lower levels of *Nox1* than *H. pylori* infected *InsGAS* mice. Results from prior studies using reductionist models (e.g., AGS cells^38^ or gastroids^32^) are expected to be vastly different in a chronic infection model with infiltration of a number of immune cells which contribute strongly to Nox isotype expression (i.e. myeloid cells). In light of the increased chronic inflammation we observe in *InsGAS*^*tg/tg*^*Il17ra*^*-/-*^, *Nox1* may be activated by increased antigen load, or by other highly expressed cytokines, such as IFNγ or TNF. Even though increases in oxidative stress can contribute to carcinogenesis, *Nox1* expression is not associated with any protective effects, but rather may potentiate *H. pylori* infection and immune activation in this *InsGAS* model. In fact, oxidative stress may be generated in the IL-17RA deficient *InsGAS* mice through a different NADPH oxidase. Our analysis suggests that the Nox2 encoding gene, *Cybb*, is significantly upregulated in the absence of IL-17RA. Increased Nox2 could drive ROS especially in myeloid cells and lead to local DNA damage perpetuating the immunopathological response to *H. pylori*. Increased *Cybb* expression does correlate with chronic inflammation, severity of disease, and expression of *Ifng*, a known activator of *Cybb*. This pathway may be responsible for the increased pH2a× and DNA damage observed in IL-17RA deficient mice.

In this *InsGAS*^*tg/tg*^*Il17ra*^*-/-*^ model of *H. pylori* infection, increases in inflammation are strongly associated with carcinogenesis. The difficulty is in pinpointing the factors that drive the increases in T cell and B cell activation in the absence of IL-17RA. Several components of the innate barrier function are altered in the *InsGAS*^*tg/tg*^*Il17ra*^*-/-*^ compared to *InsGAS*^*tg/tg*^ mice, including components of calprotectin, pIgR, and mucin. While it is highly plausible that changes in these barrier components are what drive increased *H. pylori* colonization early in the absence of IL-17RA and contribute to increased immune activation, it is difficult to adequately address this experimentally. The roles of many of these components have been assessed in *H. pylori* induced gastritis models in mice (but not in the cancer models), and while there is a clear role of each component in gastric epithelial function, it remains unclear if the loss of any individual components leads to hyperinflammation or dysplasia. Cumulative disruption of multiple components may be necessary to perpetuate chronic inflammation observed in IL-17RA-deficient mice.

The disruption in mucin production, including Muc5ac and Muc6, may reflect changes to the specialized epithelial cells of the gastric mucosa during *H. pylori* infection. A limitation of this study is that as the inflammation develops in the IL-17RA deficient mice, large numbers of B cells and T cells infiltrate the tissue, and the tissue architecture becomes very disorganized, making it more difficult to quantify the specialized epithelial cells. If there is a reduction in Muc5ac producing mucous pit cells, it is not evident in the H&E-stained sections. However, there is a clear reduction in mucin production based not only on transcript levels, but also at the protein level (immunohistochemistry for Muc5ac, Figure 3). In the intestines, it was recently demonstrated that IL-17a can activate the transcription factor Atoh1 in Lgr5+ stem cells, which in turn impacts secretory cell numbers including Paneth, tuft, goblet, and enteroendocrine cells during homeostasis and in recovery after injury.^60^ In the *InsGAS* model, there is no detectible difference in Muc5ac or Muc6 expression prior to *H. pylori* infection, but after infection, the levels increase more in the *InsGAS* mice than the IL-17RA deficient *InsGAS* mice.

These data also reiterate the importance of the IL-17RA as an essential component to the down modulation of the immune response to *H. pylori*. In various mouse models, including C57Bl/6, FVB/N, and now *InsGAS*, our studies consistently highlight the crucial role of IL-17RA in preventing exacerbated inflammation and the development of lymphoid follicles.^21–23^ While *Il17ra*^*-/-*^ mice have been employed in many infection models, including those involving gastrointestinal infections, this represents one of the few instances where inflammation levels are notably elevated. This phenotype indicates that IL-17RA may have a unique role in the stomach pathophysiology and/or it could reflect the pathogenic potential of *H. pylori* and route of antigenic uptake that occurs during this chronic bacterial infection. Th17 responses have also been shown to contribute to neurological inflammatory pathways.^61,62^ Interestingly, in experimental autoimmune encephalomyelitis (EAE), the most common experimental model for multiple sclerosis, IL-17 deficiency (modeled either in *Il17ra*^*-/-*^, *Il17a*^*-/-*^ or even *Il23*^*-/-*^) leads to a deficiency in B cell responses due to the failure of fibroblastic reticular cells in lymph nodes to undergo a metabolic switch and survive to support expansion of antigen specific B cells.^62^ This is further evidence that what we observe in the *H. pylori* model is highly specific to the gastric immune response. While both *H. pylori* and EAE models develop antigen specific inflammatory responses, the antigens enter the host through very different routes and with distinct immune activation characteristics (i.e. pathogen associated molecular patterns).

Evidence supporting IL-17’s role in mucosal immunity and barrier integrity, particularly in the gastrointestinal tract, can be found in the literature on the use of IL-17 inhibitors in treating inflammatory diseases, underscoring its relevance to human clinical medicine. Secukinumab and brodalumab, inhibitors of IL-17 signaling, were used in a trial to treat Crohn’s disease. Patients receiving anti-IL-17 antibodies experienced worsening symptoms and the trial was terminated early.^63,64^ Many studies have suggested this is due to the impacts of IL-17 on barrier integrity and function observed in the intestines in various models (reviewed in^65^). Thus far, anti-IL-17 treatments in humans have not been associated with worse stomach pathologies, but this may be the result of the significant impact on the intestines, as it has a large surface area and a more diverse microbial community than the stomach. A call to understanding the basic biology behind cytokines and their pleotropic roles has great value as the field considers immune modulatory treatments for many human disease conditions from autoimmunity to chronic inflammation to cancer.

A body of previous publications suggest that Th17 responses and/or IL-17a responses could contribute to carcinogenesis. While the data herein indicate that IL-17 receptor A plays a protective role against excessive inflammation and reduces carcinogenesis, it is worth reviewing the data that suggest IL-17a could be detrimental. In a study of about 50 gastric cancer patients, high expression of IL-17 was associated with increased microvessel density, advanced clinical stage of tumors, and lymph node metastasis, leaving authors to hypothesize that IL-17 may promote angiogenesis in the tumor microenvironment.^50^ This has also been suggested in other studies since IL-17a can recruit neutrophils by inducing production of chemokines including IL-8, CXCL1, CXCL2 and CXCL5. There is concern that this recruitment of neutrophils to the invasive margin could promote angiogenesis.^66^ Further, IL-17A-induced VEGF upregulation and neovascularization through a Stat3-mediated signaling in AGS cells.^67^ Using a sphere formation assay, it has also been demonstrated that long term culture of AGS cells with IL-17a can induce NFκB signaling and epithelial cell growth a promote stemness.^38^ Other studies have described the usefulness of IL-17a as a clinical marker of disease, while avoiding speculation on its role in disease progression. Namely, the frequency of circulating Th22 and Th17 cells was significantly higher in stage III and IV gastric cancer patients compared to the frequency in stages I and II suggesting that these cells may be a novel clinical marker for gastric cancer.^68^ Additionally, another study found that high IL-17 levels can be a prognostic indicator for the 5-year survival rates of patients -correlating with a 47% survival rate and low IL-17 levels correlating to a 83.9% survival rate.^69^ While there is significant data to suggest that IL-17a signaling through its receptor, IL-17RA, has the potential to drive carcinogenesis, our study using IL-17RA deficient mice indicates that the absence of IL-17RA leads to accelerated loss of parietal cells and development of gastric cancer. The exacerbated inflammation in the IL-17RA-deficient *InsGAS* mice may contribute to the activation of pro-carcinogenic pathways and angiogenesis. For example, IL-21 is over expressed in gastric tissue of *InsGAS*^*tg/tg*^*Il17ra*^*-/-*^ and is known to activate STAT3 in many cell types promoting the production matrix metalloproteases in human epithelial cells.^70^ Interferon gamma (*Ifng*) is also highly upregulated in our model (as well as many interferon inducible genes including chemokines) and IFNγ has been shown to induce gastric cancer cell proliferation and metastasis through NFkB signaling.^71^ Further, the antimicrobial activities of IFNγ are often facilitated by induction of superoxide generation during respiratory burst of neutrophils and macrophages, primarily via activation of nicotinamide adenine dinucleotide phosphate (NADPH) oxidases (NOXs)- including NOX2 which is encoded by *Cybb*. Our data suggest a link between *Cybb* expression, chronic inflammation, and gastric disease outcomes in the IL-17RA deficient *InsGAS* mice. However, IFNγ like IL-17a seems to have a paradoxical role in carcinogenesis. IFNγ can contribute to increased anti-tumor activity of CD8^+^ T cell response and the DNA damage response induced by NOX4 can lead to activation of cell cycle checkpoints that arrest proliferation and/or induce apoptosis of tumor cells. In our model, based on gene expression analyses, there is no evidence of a change in CD8^+^ T cell responses or *Nox4* activation in the gastric tissues of *InsGAS*^*tgtg*^*Il17ra*^*-/-*^ mice. Therefore, in this context, IFNγ may be contributing to chronic inflammation, ROS production, and carcinogenesis, but further studies would be required to confirm if this is *the* cancer driving pathway.

While IL-17a may contribute to angiogenesis and metastasis in advanced stages, the clear role of IL-17RA in preventing exacerbated inflammation during *H. pylori* infection remains evident. Clinically this is relevant because higher IL-17RA expression is a favorable prognostic marker in gastric cancer. Therefore, understanding the context in which IL-17 signaling through IL-17RA may be protective is critical as we consider immunotherapeutics for the treatment of cancer and other inflammatory diseases. Furthermore, these data build upon the field’s understanding of how chronic inflammation could also contribute to regulation of NADPH oxidases and DNA damage. In fact, *Cybb* was one of only 18 genes identified and ranked in the top 3 as a potential biomarker in gastric cancer in a recent study that utilized weighted gene co-expression network analysis and samples from The Cancer Genome Atlas (GEO, GSE13911).^72^ Additionally, studies in other cancer models suggest it is a potential therapeutic target.^56,73,74^

The IL-17RA receptor is on many cell types including epithelial cells, fibroblasts, and subsets of white blood cells. The literature suggests that its impacts on epithelial cells and fibroblasts could be major contributing factors to the control of the microbiota, inflammation, and carcinogenesis. In terms of *H. pylori* infection, we and others, have demonstrated a clear role for IL-17RA in epithelial cell biology, but have found minimal direct impacts of IL-17RA on T cell biology^23^ or B cell biology (unpublished observations) during *in vivo H. pylori* infections. Our research using the Cre-flox model, *Foxa3*^*cre*^*Il17ra*^*fl/fl*^, suggests that loss of IL-17RA on epithelial cells but not fibroblasts does lead to increased gastritis,^32^ but the impact is not as dramatic as a germline mutation in *Il17ra*. This suggests that there may be a need for more than one cell type to lose IL-17RA signaling for severe inflammation and dysplasia to develop. For example, in that model, the fibroblasts express chemokines that recruit neutrophils, contributing to partial repair of the mucosal response.^32^ Further research is needed to understand the complex interactions between IL-17RA and its ligands in fibroblasts and epithelial cells in gastric cancer. In this model, the relative contribution of exacerbated expression of other cytokines, such as IFNγ and TNF, and their effects on myeloid cells, fibroblasts, and epithelial cells are particularly relevant.

In summary, IL-17RA is essential for limiting the hyperproliferation of Th1 and Th17 responses, as well as *H. pylori*-specific B cell responses. While difficult to directly measure barrier function in the gastric tissue due to excessive inflammation, our data suggest that early deficiencies in components of innate barrier function including neutrophil recruitment and mucins, likely contribute to the increased antigen uptake and immune system activation. This chronic inflammation in the face of IL-17RA deficiency is associated with increases activated Th1 and Th17-associated cytokines and increases in the NOX2-mediated oxidative stress pathway and DNA damage. Ultimately, the data presented herein suggest that IL-17RA plays a regulatory role in the gastric mucosa during *H. pylori* infection, mitigating the development of gastric pathologies including the lymphoid follicles, dysplasia, and cancer.

## Supplementary Material

Supplemental Material

## Data Availability

Most of the data generated in this study are available within the article and its supplementary data files. An exception would be the raw data from the Nanostring IO 360 PanCancer panel which is available in the Gene Expression Omnibus (GSE281761).

## Abbreviations

IL-17RA: Interleukin 17 Receptor A
IgA: Immunoglobulin A
H. pylori: Helicobacter pylori
pIgR: polymeric immunoglobulin receptor

## References

[1] Parswelshonnet J. Bacterial infection as a cause of cancer. Environ Health Perspect. 1995;103 Suppl Nov. 8(Suppl 8):263–26. doi:10.1289/ehp.95103s8263. PMC1518971 8741796

[2] de Martel C, Georges D, Bray F, Ferlay J, Clifford GM. Global burden of cancer attributable to infections in 2018: a worldwide incidence analysis. Lancet Glob Health. 2020 Feb. 8(2):e180–e190. doi:10.1016/S2214-109X(19)30488-7. 31862245

[3] Hooi JKY, Lai WY, Ng WK, Suen MMY, Underwood FE, Tanyingoh D, Malfertheiner P, Graham DY, Wong VWS, Wu JCY, et al. Global prevalence of Helicobacter pylori infection: systematic review and meta-analysis. Gastroenterology. 2017 Aug. 153(2):420–429. doi:10.1053/j.gastro.2017.04.022. 28456631

[4] FitzGerald R, Smith SM. An overview of Helicobacter pylori infection. In: Smith S, editor. Helicobacter Pylori. Humana (NY): Springer US; 2021. p. 1–14. 10.1007/978-1-0716-1302-3_1 33765303

[5] Gu J, He F, Clifford GM, Li M, Fan Z, Li X, Wang S, Wei W. A systematic review and meta-analysis on the relative and attributable risk of Helicobacter pylori infection and cardia and non-cardia gastric cancer. Expert Rev Mol Diagn. 2023 Jul. 23(12):1251–1261. doi:10.1080/14737159.2023.2277377. 37905778

[6] Correa P, Houghton J. Carcinogenesis of Helicobacter pylori. Gastroenterology. 2007 Aug. 133(2):659–672. doi:10.1053/j.gastro.2007.06.026. 17681184

[7] Correa P, Piazuelo MB, Wilson KT. Pathology of gastric intestinal metaplasia: clinical implications. Am J Gastroenterol. 2010 Mar. 105(3):493–498. doi:10.1038/ajg.2009.728. 20203636 PMC2895407

[8] Bray F, Laversanne M, Sung H, Ferlay J, Siegel RL, Soerjomataram I, Jemal A. Global cancer statistics 2022: GLOBOCAN estimates of incidence and mortality worldwide for 36 cancers in 185 countries. CA Cancer J Clin. 2024 May. 74(3):229–263. doi:10.3322/caac.21834. 38572751

[9] SEER*Explorer: An interactive website for SEER cancer statistics [Internet]. Surveillance Research Program, National Cancer Institute; 2024 Apr 17. [updated: 2024 Nov 5; cited 2024 Nov 18]. Available from: https://seer.cancer.gov/statistics-network/explorer/. Data source(s): SEER Incidence Data, November 2023 Submission (1975-2021), SEER 22 registries.

[10] Malfertheiner P, Camargo MC, El-Omar E, Liou J-M, Peek R, Schulz C, Smith SI, Suerbaum S. Helicobacter pylori infection. Nat Rev Dis Primers. 2023/04/20 2023;9(1):19. doi:10.1038/s41572-023-00431-8. 37081005 PMC11558793

[11] Nie S, Yuan Y. The role of gastric mucosal immunity in gastric diseases. J Immunol Res. 2020;2020:7927054. doi:10.1155/2020/7927054. 32775468 PMC7396052

[12] Jafarzadeh A, Larussa T, Nemati M, Jalapour S. T cell subsets play an important role in the determination of the clinical outcome of Helicobacter pylori infection. Microb Pathog. 2018 Mar. 116:227–236. doi:10.1016/j.micpath.2018.01.040. 29407232

[13] Larussa T, Leone I, Suraci E, Imeneo M, Luzza F. Helicobacter pylori and T helper cells: mechanisms of immune escape and tolerance. J Immunol Res. 2015;2015:981328. doi:10.1155/2015/981328. 26525279 PMC4615206

[14] Algood HMS. T cell cytokines impact epithelial cell responses during Helicobacter pylori infection. The J Immunol. [2020 Mar 15]. 204(6):1421–1428. doi:10.4049/jimmunol.1901307. 32152211 PMC7080313

[15] Eck M, Schmausser B, Scheller K, Toksoy A, Kraus M, Menzel T, Müller-Hermelink HK, Gillitzer R. CXC chemokines gro α /IL-8 and IP-10/MIG in Helicobacter pylori gastritis. Clin Exp Immunol. 2000 Nov. 122(2):192–199. doi:10.1046/j.1365-2249.2000.01374.x. 11091274 PMC1905774

[16] Morey P, Pfannkuch L, Pang E, Boccellato F, Sigal M, Imai-Matsushima A, Dyer V, Koch M, Mollenkopf H-J, Schlaermann P, et al. Helicobacter pylori depletes cholesterol in gastric glands to prevent interferon gamma signaling and escape the inflammatory response. Gastroenterology. 2018 Apr. 154(5):1391–1404 e9. doi:10.1053/j.gastro.2017.12.008. 29273450

[17] Caruso R, Fina D, Paoluzi OA, Del vecchio blanco G, Stolfi C, Rizzo A, Caprioli F, Sarra M, Andrei F, Fantini M, et al. IL-23-mediated regulation of IL-17 production in Helicobacter pylori-infected gastric mucosa. Eur J Immunol. 2008 Feb. 38(2):470–478. doi:10.1002/eji.200737635. 18200634

[18] Algood HM, Gallo-Romero J, Wilson KT, Peek RM Jr., Cover TL. Host response to Helicobacter pylori infection before initiation of the adaptive immune response. FEMS Immunol and Med Microbiol. 2007 Dec. 51(3):577–586. doi:10.1111/j.1574-695X.2007.00338.x. 17919297

[19] Sugimoto M, Ohno T, Graham DY, Yamaoka Y. Gastric mucosal interleukin-17 and -18 mRNA expression in Helicobacter pylori-induced Mongolian gerbils. Research support, N.I.H. extramural research support, U.S. Gov’t, non-P.H.S. Cancer Sci. Nov 2009;100(11):2152–2159. doi:10.1111/j.1349-7006.2009.01291.x. 19694753 PMC3128813

[20] Horvath DJ Jr., Washington MK, Cope VA, Algood HM. IL-23 contributes to control of chronic Helicobacter Pylori infection and the development of T helper responses in a mouse Model. Front Immun. 2012;3:56. doi:10.3389/fimmu.2012.00056. PMC3342083 22566937

[21] Algood HM, Allen SS, Washington MK, Peek RM Jr., Miller GG, Cover TL. Regulation of gastric B cell recruitment is dependent on IL-17 receptor a signaling in a model of chronic bacterial infection. The J Immunol. [2009 Nov 1]. 183(9):5837–5846. doi:10.4049/jimmunol.0901206. 19812196 PMC2834183

[22] Brackman LC, Dixon B, Bernard M, Revetta F, Cowell RP, Meenderink LM, Washington MK, Piazuelo MB, Algood HMS. IL-17 receptor a functions to help maintain barrier integrity and limit activation of immunopathogenic response to H. pylori infection. Infect Immun. [2024 Jan 16]. 92(1):e0029223. doi:10.1128/iai.00292-23. 38014948 PMC10790819

[23] Dixon B, Lee TJ, Contreras Healey DC, Li J, Goettel JA, Piazuelo MB, Algood HMS. IL-17 receptor signaling through IL-17A or IL-17F is sufficient to maintain innate response and control of Helicobacter pylori Immunopathogenesis. Immunohorizons. [2022 Feb 10]. 6(2):116–129. doi:10.4049/immunohorizons.2000072. 35144998 PMC9364271

[24] Gaffen SL. Recent advances in the IL-17 cytokine family. Curr Opin Immunol. 2011 Oct. 23(5):613–619. doi:10.1016/j.coi.2011.07.006. 21852080 PMC3190066

[25] Miossec P, Korn T, Kuchroo VK. Interleukin-17 and type 17 helper T cells. N Engl J Med. [2009 Aug 27]. 361(9):888–898. doi:10.1056/NEJMra0707449. 19710487

[26] Alam MS, Costales M, Cavanaugh C, Pereira M, Gaines D, Williams K. Oral exposure to listeria monocytogenes in aged IL-17RKO mice: a possible murine model to study listeriosis in susceptible populations. Microb Pathog. 2016 Oct. 99:236–246. doi:10.1016/j.micpath.2016.08.035. 27574777

[27] Conti HR, Shen F, Nayyar N, Stocum E, Sun JN, Lindemann MJ, Ho AW, Hai JH, Yu JJ, Jung JW, et al. Th17 cells and IL-17 receptor signaling are essential for mucosal host defense against oral candidiasis. J Exp Med. [2009 Feb 16]. 206(2):299–311. doi:10.1084/jem.20081463. 19204111 PMC2646568

[28] Dreesen L, De Bosscher K, Grit G, Staels B, Lubberts E, Bauge E, Geldhof P. Giardia muris infection in mice is associated with a protective interleukin 17A response and induction of peroxisome proliferator-activated receptor alpha. Infect Immun. 2014 Aug. 82(8):3333–3340. doi:10.1128/IAI.01536-14. 24866800 PMC4136230

[29] Ye P, Rodriguez FH, Kanaly S, Stocking KL, Schurr J, Schwarzenberger P, Oliver P, Huang W, Zhang P, Zhang J, et al. Requirement of interleukin 17 receptor signaling for lung CXC chemokine and granulocyte colony-stimulating factor expression, neutrophil recruitment, and host defense. J Exp Med. [2001 Aug 20]. 194(4):519–527. doi:10.1084/jem.194.4.519. 11514607 PMC2193502

[30] Human Protein Atlas. Updated. 2023 June 19 [accessed 2023 Dec 1]. https://www.proteinatlas.org/ENSG00000177663-IL17RA/pathology.

[31] Uhlen M, Fagerberg L, Hallstrom BM, Lindskog C, Oksvold P, Mardinoglu A, Sivertsson Å, Kampf C, Sjöstedt E, Asplund A, et al. Proteomics. Tissue-based map of the human proteome. Science. [2015 Jan 23]. 347(6220):1260419. doi:10.1126/science.1260419. 25613900

[32] Brackman LC, Jung MS, Ogaga EI, Joshi N, Wroblewski LE, Piazuelo MB, Peek RM, Choksi YA, Algood HMS. IL-17RA–mediated epithelial cell activity prevents severe inflammatory response to Helicobacter pylori infection. Immunohorizons. [2024 Apr 1]. 8(4):339–353. doi:10.4049/immunohorizons.2300078. 38639570 PMC11066722

[33] Hou C, Yang F. Interleukin-17A gene polymorphism is associated with susceptibility to gastric cancer. Int J Clin Exp Pathol. 2015;8:7378–7384. 26261639 PMC4525973

[34] Liu J, Xu Q, Yuan Q, Wang Z, Xing C, Yuan Y. Association of IL-17A and IL-17F polymorphisms with gastric cancer risk in asians: a meta-analysis. Hum Immunol. 2015 Jan. 76(1):6–12. doi:10.1016/j.humimm.2014.12.011. 25500254

[35] Qinghai Z, Yanying W, Yunfang C, Xukui Z, Xiaoqiao Z. Effect of interleukin-17A and interleukin-17F gene polymorphisms on the risk of gastric cancer in a Chinese population. Gene. [2014 Mar 10]. 537(2):328–332. doi:10.1016/j.gene.2013.11.007. 24315816

[36] Rafiei A, Hosseini V, Janbabai G, Ghorbani, A, Ajami, A, Farzmandfar, T, Azizi, MD, Gilbreath, JJ, Merrell, DS. Polymorphism in the interleukin-17A promoter contributes to gastric cancer. World J Gastroenterol. [2013 Sep 14]. 19(34):5693–5699. doi:10.3748/wjg.v19.i34.5693. 24039363 PMC3769907

[37] Su Z, Sun Y, Zhu H, Liu Y, Lin X, Shen H, Chen J, Xu W, Xu H. Th17 cell expansion in gastric cancer may contribute to cancer development and metastasis. Immunol Res. 2014 Jan. 58(1):118–124. doi:10.1007/s12026-013-8483-y. 24402773

[38] Kang JH, Park S, Rho J, Hong E-J, Cho Y-E, Won Y-S, Kwon H-J. IL-17A promotes Helicobacter pylori-induced gastric carcinogenesis via interactions with IL-17RC. Gastric Cancer. 2023 Jan. 26(1):82–94. doi:10.1007/s10120-022-01342-5. 36125689 PMC9813207

[39] Iida T, Iwahashi M, Katsuda M, Ishida, K, Nakamori, M, Nakamura, M, Naka, T, Ojima, T, Ueda, K, Hayata, K, et al. Tumor-infiltrating CD4+ Th17 cells produce IL-17 in tumor microenvironment and promote tumor progression in human gastric cancer. Oncol Rep. 2011 May. 25(5):1271–1277. doi:10.3892/or.2011.1201. 21369705

[40] Wang TC, Bonner-Weir S, Oates PS, Chulak M, Simon B, Merlino GT, Schmidt EV, Brand SJ. Pancreatic gastrin stimulates islet differentiation of transforming growth factor alpha-induced ductular precursor cells. J Clin Invest. 1993 Sep. 92(3):1349–1356. doi:10.1172/JCI116708. 8376589 PMC288276

[41] Perkins JR, Dawes JM, Sb M, Bennett DL, Orengo C, Kohl M. ReadqPCR and NormqPCR: R packages for the reading, quality checking and normalisation of rt-qPCR quantification cycle (cq) data. BMC Genomics. [2012 Jul 2]. 13(1):296. doi:10.1186/1471-2164-13-296. 22748112 PMC3443438

[42] Jiang Y, Yu Y. Transgenic and gene knockout mice in gastric cancer research. Oncotarget. [2017 Jan 10]. 8(2):3696–3710. doi:10.18632/oncotarget.12467. 27713138 PMC5356912

[43] Lee CW, Rickman B, Rogers AB, Ge Z, Wang TC, Fox JG. Helicobacter pylori eradication prevents progression of gastric cancer in hypergastrinemic INS-GAS mice. Cancer Res. [2008 May 1]. 68(9):3540–3548. doi:10.1158/0008-5472.CAN-07-6786. 18441088 PMC2653414

[44] Stewart OA, Wu F, Chen Y. The role of gastric microbiota in gastric cancer. Gut Microbes. [2020 Sep 2]. 11(5):1220–1230. doi:10.1080/19490976.2020.1762520. 32449430 PMC7524314

[45] Fox JG, Wang TC, Rogers AB, Poutahidis T, Ge Z, Taylor N, Dangler CA, Israel DA, Krishna U, Gaus K, et al. Host and microbial constituents influence Helicobacter pylori-induced cancer in a murine model of hypergastrinemia. Gastroenterology. 2003 June. 124(7):1879–1890. doi:10.1016/s0016-5085(03)00406-2. 12806621

[46] Wang TC, Dangler CA, Chen D, Goldenring JR, Koh T, Raychowdhury R, Coffey RJ, Ito S, Varro A, Dockray GJ, et al. Synergistic interaction between hypergastrinemia and Helicobacter infection in a mouse model of gastric cancer. Gastroenterology. 2000 Jan. 118(1):36–47. doi:10.1016/s0016-5085(00)70412-4. 10611152

[47] Fox JG, Rogers AB, Ihrig M, Taylor NS, Whary MT, Dockray G, Varro A, Wang TC. Helicobacter pylori-associated gastric cancer in INS-GAS mice is gender specific. Cancer Res. [2003 Mar 1]. 63(5):942–950. 12615707

[48] Hayakawa Y, Fox JG, Gonda T, Worthley DL, Muthupalani S, Wang TC. Mouse models of gastric cancer. Cancers (Basel). [2013 Jan 24]. 5(1):92–130. doi:10.3390/cancers5010092. 24216700 PMC3730302

[49] Poh AR, Rj O, Ernst M, Putoczki TL. Mouse models for gastric cancer: matching models to biological questions. J Gastroenterol Hepatol. 2016 Jul. 31(7):1257–1272. doi:10.1111/jgh.13297. 26809278 PMC5324706

[50] Meng XY, Zhou CH, Ma J, Jiang C, Ji P. Expression of interleukin-17 and its clinical significance in gastric cancer patients. Med Oncol. 2012 Dec. 29(5):3024–3028. doi:10.1007/s12032-012-0273-1. 22744708

[51] Tiburca L, Bembea M, Zaha DC, Jurca AD, Vesa CM, Rațiu IA, Jurca CM. The treatment with interleukin 17 inhibitors and immune-mediated inflammatory diseases. Curr Issues Mol Biol. [2022 Apr 26]. 44(5):1851–1866. doi:10.3390/cimb44050127. 35678656 PMC9164043

[52] Song M, Liang J, Wang L, Li W, Jiang S, Xu S, Tang L, Du Q, Liu G, Meng H, et al. IL-17A functions and the therapeutic use of IL-17A and IL-17RA targeted antibodies for cancer treatment. Int Immunopharmacol. 2023 Oct. 123:110757. doi:10.1016/j.intimp.2023.110757. 37579542

[53] Cao AT, Yao S, Gong B, Elson CO, Cong Y. Th17 cells upregulate polymeric ig receptor and intestinal IgA and contribute to intestinal homeostasis. The J Immunol. [2012 Nov 1]. 189(9):4666–4673. doi:10.4049/jimmunol.1200955. 22993206 PMC3478497

[54] Frazao JB, Thain A, Zhu Z, Luengo M, Condino-Neto A, Newburger PE. Regulation of CYBB gene expression in human phagocytes by a distant upstream nf-kappaB binding site. J Cell Biochem. 2015 Sep. 116(9):2008–2017. doi:10.1002/jcb.25155. 25752509 PMC5551681

[55] Wang P, Shi Q, Deng WH, Yu, J, Zuo, T, Mei, FC, Wang, WX. Relationship between expression of NADPH oxidase 2 and invasion and prognosis of human gastric cancer. World J Gastroenterol. [2015 May 28]. 21(20):6271–6279. doi:10.3748/wjg.v21.i20.6271. 26034362 PMC4445104

[56] Grauers Wiktorin H, Aydin E, Hellstrand K, Martner A. NOX2-derived reactive oxygen species in cancer. Oxid Med Cell Longev. 2020;2020:7095902. doi:10.1155/2020/7095902. 33312338 PMC7721506

[57] Gobert AP, Asim M, Smith TM, Williams KJ, Barry DP, Allaman MM, McNamara KM, Hawkins CV, Delgado AG, Blanca Piazuelo M, et al. The nutraceutical electrophile scavenger 2-hydroxybenzylamine (2-HOBA) attenuates gastric cancer development caused by Helicobacter pylori. Biomed Pharmacother. 2023 Feb. 158:114092. doi:10.1016/j.biopha.2022.114092. 36493697 PMC9879697

[58] Sierra JC, Asim M, Verriere TG, Piazuelo MB, Suarez G, Romero-Gallo J, Delgado AG, Wroblewski LE, Barry DP, Peek RM, et al. Epidermal growth factor receptor inhibition downregulates Helicobacter pylori-induced epithelial inflammatory responses, DNA damage and gastric carcinogenesis. Gut. 2018 Jul. 67(7):1247–1260. doi:10.1136/gutjnl-2016-312888. 28473630 PMC5671361

[59] Li X, Bechara R, Zhao J, Mj M, Gaffen SL. IL-17 receptor-based signaling and implications for disease. Nat Immunol. 2019 Dec. 20(12):1594–1602. doi:10.1038/s41590-019-0514-y. 31745337 PMC6943935

[60] Lin X, Gaudino SJ, Jang KK, Bahadur T, Singh A, Banerjee A, Beaupre M, Chu T, Wong HT, Kim C-K, et al. IL-17RA-signaling in Lgr5(+) intestinal stem cells induces expression of transcription factor ATOH1 to promote secretory cell lineage commitment. Immunity. [2022 Feb 8]. 55(2):237–253 e8. doi:10.1016/j.immuni.2021.12.016. 35081371 PMC8895883

[61] Peters A, Pitcher LA, Sullivan JM, Mitsdoerffer M, Acton S, Franz B, Wucherpfennig K, Turley S, Carroll M, Sobel R, et al. Th17 cells induce ectopic lymphoid follicles in central nervous system tissue inflammation. Immunity. [2011 Dec 23]. 35(6):986–996. doi:10.1016/j.immuni.2011.10.015. 22177922 PMC3422678

[62] Majumder S, Amatya N, Revu S, Jawale CV, Wu D, Rittenhouse N, Menk A, Kupul S, Du F, Raphael I, et al. IL-17 metabolically reprograms activated fibroblastic reticular cells for proliferation and survival. Nat Immunol. 2019 May. 20(5):534–545. doi:10.1038/s41590-019-0367-4. 30962593 PMC6519710

[63] Hueber W, Sands BE, Lewitzky S, et al. Secukinumab, a human anti-IL-17A monoclonal antibody, for moderate to severe Crohn’s disease: unexpected results of a randomised, double-blind placebo-controlled trial. Gut. 2012 Dec. 61(12):1693–1700. doi:10.1136/gutjnl-2011-301668. 22595313 PMC4902107

[64] Targan SR, Feagan B, Vermeire S, Panaccione R, Melmed GY, Landers C, Li D, Russell C, Newmark R, Zhang N, et al. A randomized, double-blind, placebo-controlled phase 2 study of Brodalumab in patients with moderate-to-severe Crohn’s disease. Am J Gastroenterol. 2016 Nov. 111(11):1599–1607. doi:10.1038/ajg.2016.298. 27481309

[65] Huangfu L, Li R, Huang Y, Wang S. The IL-17 family in diseases: from bench to bedside. Signal Transduct Target Ther. [2023 Oct 11]. 8(1):402. doi:10.1038/s41392-023-01620-3. 37816755 PMC10564932

[66] Murdoch C, Muthana M, Coffelt SB, Lewis CE. The role of myeloid cells in the promotion of tumour angiogenesis. Nat Rev Cancer. 2008 Aug. 8(8):618–631. doi:10.1038/nrc2444. 18633355

[67] Wu X, Yang T, Liu X, Guo JN, Xie T, Ding Y, Lin M, Yang H. IL-17 promotes tumor angiogenesis through Stat3 pathway mediated upregulation of VEGF in gastric cancer. Tumour Biol. 2016 Apr. 37(4):5493–5501. doi:10.1007/s13277-015-4372-4. 26566627

[68] Liu T, Peng L, Yu P, Zhao Y, Shi Y, Mao X, Chen W, Cheng P, Wang T, Chen N, et al. Increased circulating Th22 and Th17 cells are associated with tumor progression and patient survival in human gastric cancer. J Clin Immunol. 2012 Dec. 32(6):1332–1339. doi:10.1007/s10875-012-9718-8. 22760549

[69] Yamada Y, Saito H, Ikeguchi M. Prevalence and clinical relevance of Th17 cells in patients with gastric cancer. J Surg Res. 2012 Dec. 178(2):685–691. doi:10.1016/j.jss.2012.07.055. 22940035

[70] Ming S, Yin H, Li X, Gong S, Zhang G, Wu Y. GITR promotes the polarization of TFH-Like cells in Helicobacter pylori-positive gastritis. Front Immunol. 2021;12:736269. doi:10.3389/fimmu.2021.736269. 34589088 PMC8475268

[71] Xu YH, Li ZL, Qiu SF. Ifn-gamma induces gastric cancer cell proliferation and metastasis through upregulation of integrin beta3-mediated nf-kappaB signaling. Transl Oncol. 2018 Feb. 11(1):182–192. doi:10.1016/j.tranon.2017.11.008. 29306706 PMC5755748

[72] Chen H, Sun Q, Zhang C, She J, Cao S, Cao M, Zhang N, Adiila AV, Zhong J, Yao C, et al. Identification and validation of CYBB, CD86, and C3AR1 as the key genes related to macrophage infiltration of gastric cancer. Front Mol Biosci. 2021;8:756085. doi:10.3389/fmolb.2021.756085. 34950700 PMC8688826

[73] Wang Y, Wang Y, Wang S, Wang C, Tang Y, Zhang C, Yu D, Hou S, Lin N. Comprehensive analysis of CYBB as a prognostic marker and therapeutic target in glioma: a bioinformatics approach. Heliyon. [2024 Apr 30]. 10(8):e29549. doi:10.1016/j.heliyon.2024.e29549. 38655339 PMC11036048

[74] Bode K, Hauri-Hohl M, Jaquet V, Weyd H. Unlocking the power of NOX2: a comprehensive review on its role in immune regulation. Redox Biol. 2023 Aug. 64:102795. doi:10.1016/j.redox.2023.102795. 37379662 PMC10320620

