# Supplementary material for: IL-17 signaling protects against Helicobacter pylori-induced gastric cancer
Source: Gut Microbes. 2024 Nov 26;16(1):2430421. doi: 10.1080/19490976.2024.2430421 (PMC11639209; doi:10.1080/19490976.2024.2430421)
Supplement: Supplemental Material [file KGMI_A_2430421_SM8190.zip › SupplementalMaterials_INSGAS_GutMicro_resubmission.docx]

**Supplemental Figure 1. Female *InsGAS^tg/tg^Il17ra^-/-^* do not colonize as efficiently but develop severe pathology associated with *H. pylori* infection.** A. Colonization is determined by counting dilutions of plated stomach homogenates. A single colony was interpreted as successful colonization. Number of colonized v. non-colonized mice are represented in the graph (percentage of mice colonized is noted). Fisher’s Exact test on these data indicated that colonization was not significantly associated with sex of the mice (P value= 0.1448). B. Of the successfully colonized mice, there was no difference in the bacterial burden between groups at 6-weeks post infection. Statistical significance was tested using an unpaired t-test performed on log transformed CFU/g values. C. Total inflammation, which combines chronic and acute inflammation scores, and lymphoid follicle counts from infected mice at the 6-week time point are presented. Significance between sexes was tested using a Kruskal-Wallis test for inflammation and an unpaired t-test for lymphoid follicle counts (ns, not significant).


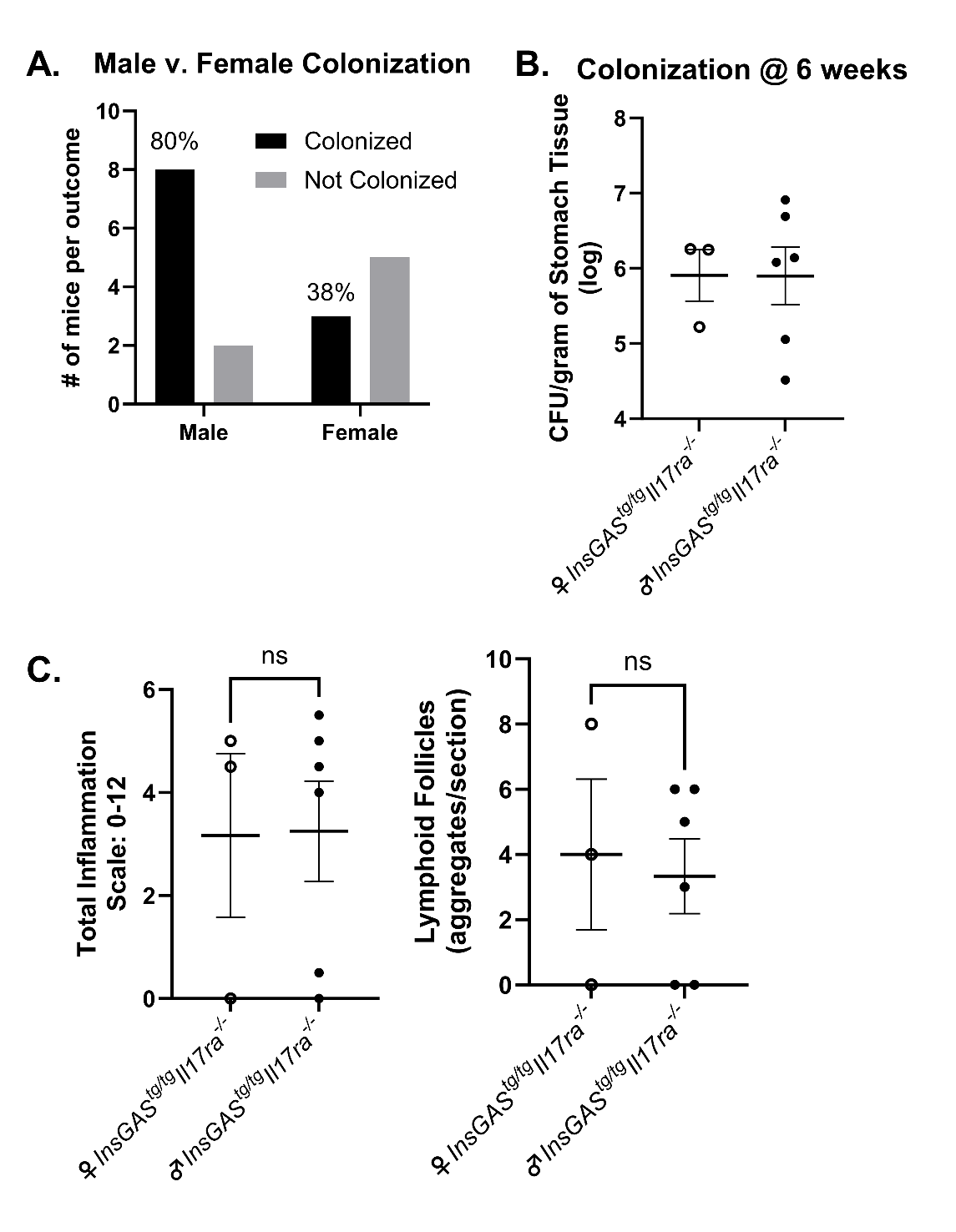


**Supplemental Figure 2. *Increased inflammation in the absence of IL-17RA is associated with changes in populations of specialized epithelial cells***. Loss of parietal cells, loss of chief cells, and foveolar hyperplasia were scored in mice infected with *H. pylori* for 6 weeks (A), 3 months (B), and 6 months (C).

Scores range from 0 (no loss of cells) to 1 (≤30% loss) to 2 (>30-60% loss) to 3 (>60% loss of cell type). Mann- Whitney U test was run to determine statistical significance. *p<0.05, **p<0.01,**p<0.001. Corpus foveolar hyperplasia was scored as none (0), mild (1), moderate (2) or severe (3).


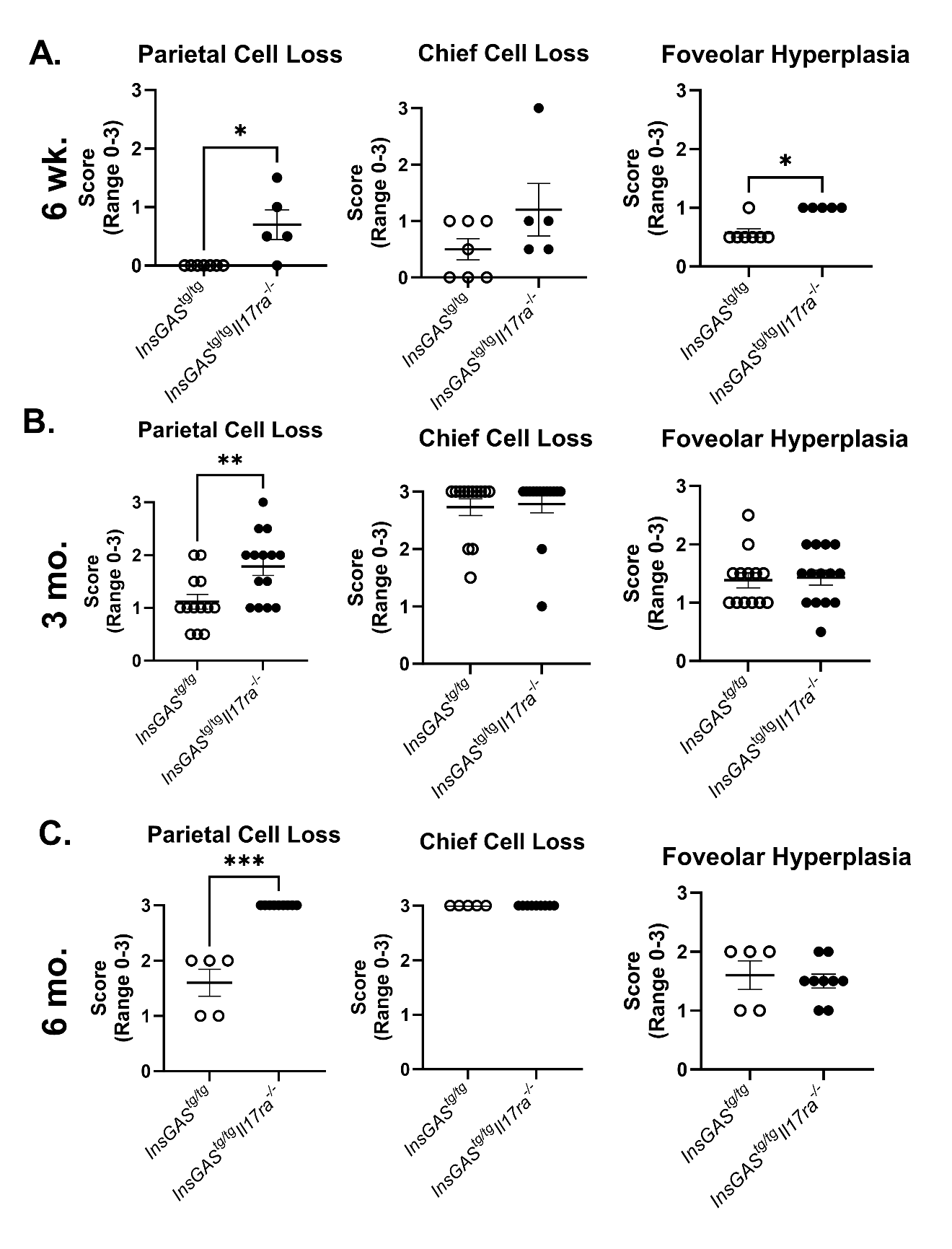


**Supplemental Figure 3. *IL-17RA is required to limit the development of dysplasia in response to H. pylori infection.*** At 6 mo. post infection, gastric tissue was collected to A. quantify CFU/g of tissue, score total inflammation, B. assess atrophy of chief cells and parietal cells, and diagnose disease. Error bars represent mean ± SEM. See Materials and Methods for the scoring systems. *p ≤ 0.05, **p ≤ 0.01, ****p ≤ 0.0001. Panel C are representative hematoxylin and eosin (H&E) stained tissues at 6 months post infection. Images are of the corpus at 100x magnification illustrating increased total inflammation and dysplasia.


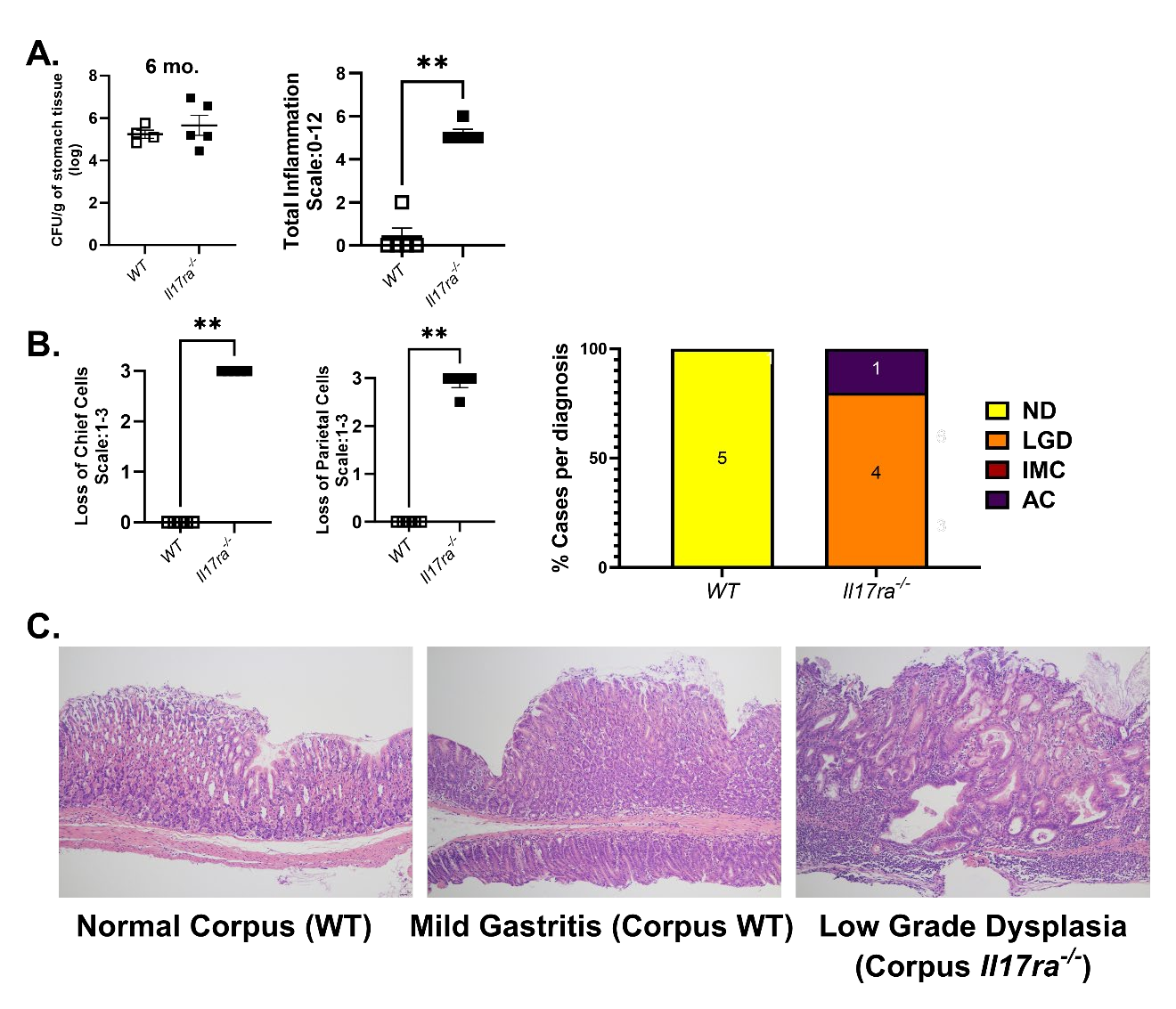


**Supplemental Figure 4. Anti-IL-17A does not impact the immunopathological response in *InsGAS* mice.**

1.
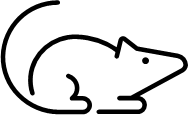
*InsGAS* mice were infected with *H. pylori* or left uninfected and then after 6 weeks, mice received either anti- IL-17A or IgG intraperitoneally, 2 times a week for 6 weeks. All mice were sacrificed and B. bacterial burden (CFU/gram of gastric tissue), the inflammatory response, and impact on stomach histology was assessed.


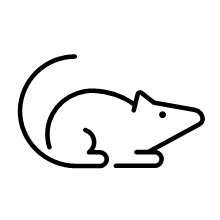

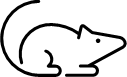

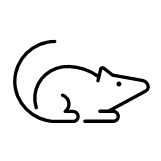

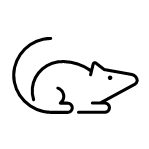

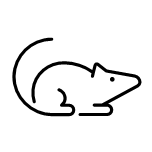

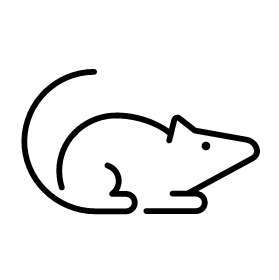


**A.**

Anti-IL17A

*Helicobacter pylori*

IgG control

8-10 weeks of age

6 weeks

Mice Sacrificed:

3 months CFU/g Histology

Tissue snap Frozen

Anti-IL17A

*Uninfected controls*

**3 months**

ns

**7**

**CFU/g of stomach tissue (log)**

**6**

**5**

**4**

**3**

IgG control

**Total Inflammation Scores**

✱✱

✱✱✱

ns

**5 4**

**# Lymphoid Follicles**

**/ Section**

**4 3**

**Total Inflammation (Scale:0-12)**

**3**

**2**

**2**

**1 1**

**Lymphoid Follicles**

**2 0 0**

uninfected 3mo. PMSS1

uninfected 3mo. PMSS1

**Supplemental Figure 5. *H. pylori* infection is required for most observed differences in gene expression in the gastric mucosa, but not all.** A. Expression of genes which impact barrier were assessed in uninfected mice including *Pigr, Muc5ac* and *Muc6* by real time rtPCR. No differences in expression levels were detected in these tissues. B. Volcano Plot of differentially expressed genes in uninfected *InsGAS^tg/tg^* stomach tissue compared to uninfected *InsGAS^tg/tg^Il17ra^-/-^* tissue (at 5 mo old). Differential gene expression was determined by analyzing data generated using the Nanostring PanCancer IO360 nCounter panel using n=5 samples per genotype. C. Expression of T helper cytokine genes were measured by real time rtPCR including *Il17a, Il21,* and *Ifng.* *p<0.05. D. Expression of *Nox1* and *Nox4* over the time course was measured by realtime rtPCR.

**A. 5 B.**

ns

ns


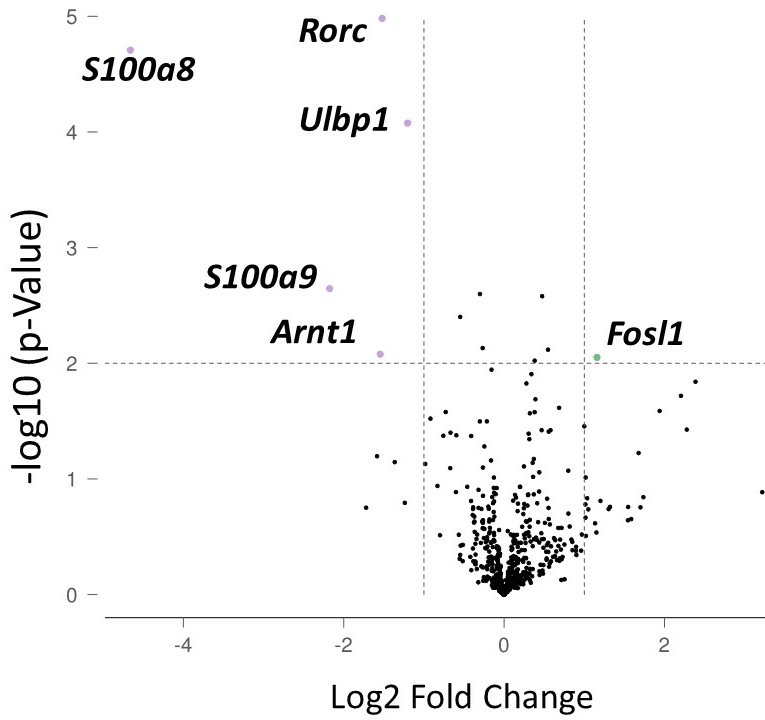


ns

**10**

**8**

**6**

**4**

✱

**2**

**0**

**5**

**5**

**4 4 4**

**Relative Units (*Muc5ac*)**

**Relative Units (*Muc6*)**

**Relative Units**

***(Pigr)***

**3 3**

**3**

**2 2**

**2**

**1 1**

**1 0 0**

1. **100 10**

✱

ns

**80 8**

**Relative Units (*Il17a*)**

**Relative Units (*Il21*)**

**Relative Units (*Ifng*)**

**60 6**

**40 4**

**20 2**

**0 0**

1. **Uninfected**

ns

**8**

**6**

**Relative Units (*Nox1*)**

**4**

**2**

**6 wk.**

**30**


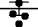


ns

**20**

**Relative Units (*Nox1*)**

**10**

**200**

**150**

**Relative Units (*Nox1*)**

**100**

**50**

**3 mo.**

**6 mo.**

**50**

ns

**40**

**Relative Units (*Nox1*)**

**30**

**20**

**10**

**0 0 0 0**

**5 8 6 wk.**


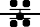

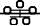


**Uninfected**

ns


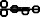


ns

**4 6**

**Relative Units (*Nox4*)**

**Relative Units (*Nox4*)**

**3 4**

**2 2**

**1 0**

**3 mo.**


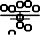


✱

**5**


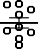


ns

**4**

**Relative Units (*Nox4*)**

**3**

**2**

**1**

**0**

**6 mo.**

**5**


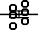


ns

**4**

**Relative Units (*Nox4*)**

**3**

**2**

**1**

**0**

**Supplemental Figure 6. Gating strategy for flow cytometry.** Forward Scatter (FSC) and Side Scatter (SSC) were used to determine the location of the target population based upon size and granularity. From that population, Viability Dye (Supplemental Figure 1) differentiates live from dead cells. We then differentiated our CD45+ population from viable cells into CD4+ (T cells) and CD45R+ (B cells).


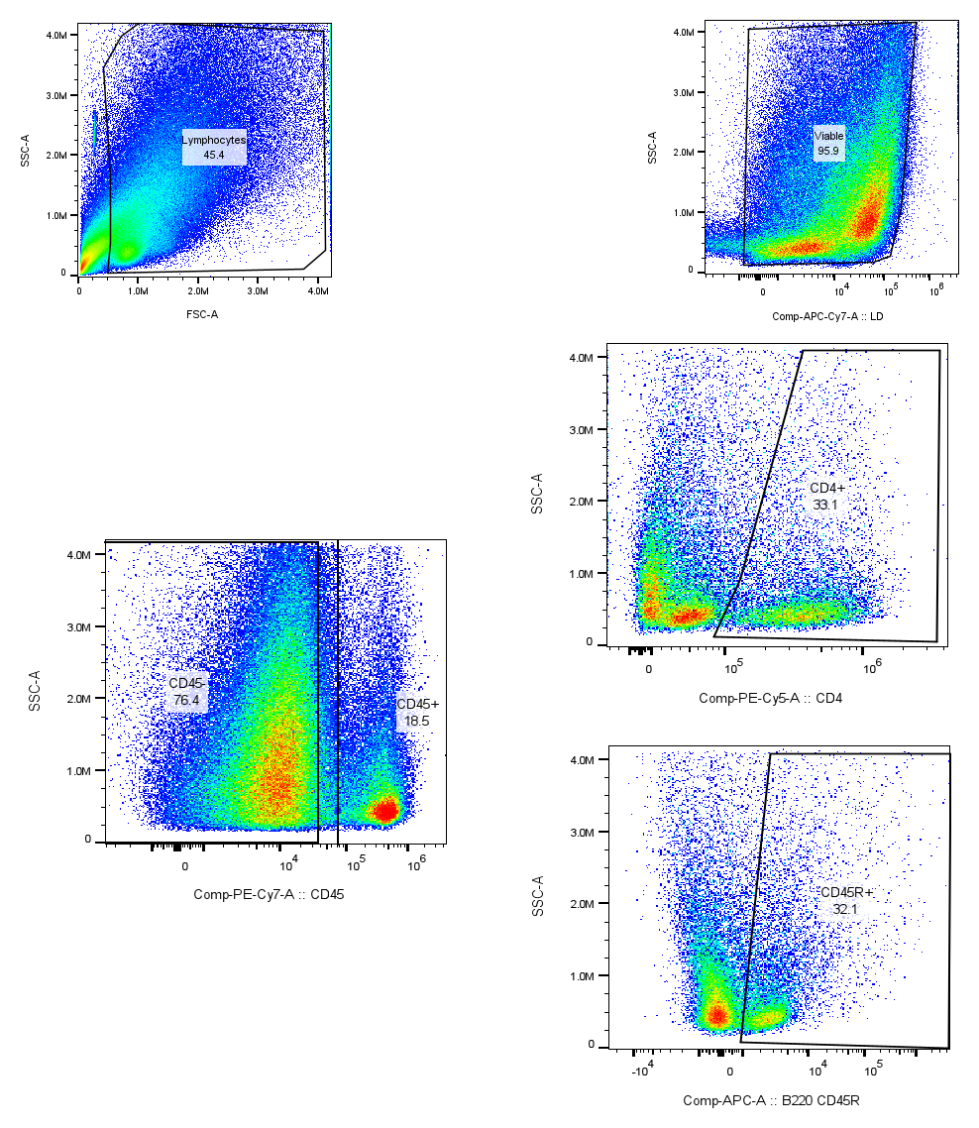


**Supplemental Table 1. Antibodies for Flow Cytometry** Antibodies, Fluorophores, Dilutions, Clones, Manufacturers, and Catalog Numbers for Antibodies used in flow cytometry experiments performed on the SpectroFlow instrument.

| ***Stains*** | ***Fluorophore*** | ***Dilution*** | ***Clone (Cat #)*** |
| --- | --- | --- | --- |
| **Anti-CD45** | PE-Cy7 | 1:750 | 30-F11 (60-0451-U025)  Tonbo Biosciences |
| **Anti-CD4** | PE-Cy5 | 1:500 | H129.19 (553654)  BD Biosciences |
| **Anti-B220 (CD45R)** | APC | 1:500 | RA3-862 (20-0452-U100)  Tonbo Biosciences |
| **Viability Dye** | eFluor780 | 1:10000 | 65-0865-14  eBioscience™ |

**Supplemental Table 2.** Differential gene expression in gastric tissue from uninfected *InsGAS^tg/tg^Il17ra^-/-^* mice relative to *InsGAS* tissue at 5 months of age. Genes with a >+/-2 fold change (>1.2 Log fold change) and a p- Value < 0.01 are on this table. Genes expression analysis was performed using Nanostring’s PanCancer IO 360 panel which contained 770 mouse genes. Analysis was performed on Rosalind as described in the *Materials* and *Methods.* (n=5 mice per genotype)

| **Gene Name** | **Description** | **Log Fold Change** | **p-Value** | **p-Adj** |
| --- | --- | --- | --- | --- |
| *Arnt2* | aryl hydrocarbon receptor nuclear translocator 2 | -1.54565 | 0.00835 | 0.455422 |
| *Fosl1* | fos-like antigen 1 | 1.15843 | 0.008901 | 0.455422 |
| *Rorc* | RAR-related orphan receptor gamma | -1.52159 | 1.04E-05 | 0.005641 |
| *S100a8* | S100 calcium binding protein A8 (calgranulin A) | -4.66131 | 1.96E-05 | 0.005641 |
| *S100a9* | S100 calcium binding protein A9 (calgranulin B) | -2.17571 | 0.002264 | 0.252617 |
| *Ulbp1* | UL16 binding protein 1 | -1.20353 | 8.38E-05 | 0.016081 |

**Supplemental Table 3**. Differential gene expression in gastric tissue from *InsGAS^tg/tg^Il17ra^-/-^* mice relative to *InsGAS* tissue at 3 months post infection. Genes with a >3 fold change (>1.59 Log fold change) and a p-Adj Value < 0.01 are on this table. Genes expression analysis was performed using Nanostring’s PanCancer IO 360 panel which contained 770 mouse genes. Analysis was performed on Rosalind as described in the *Materials* and *Methods.* (n=13 mice per genotype)

| ***Gene Name*** | **Description** | **Log Fold Change** | **p-Value** | **p-Adj** |
| --- | --- | --- | --- | --- |
| *Cxcl10* | chemokine (C-X-C motif) ligand 10 | 5.15523 | 5.74E-09 | 7.16E-08 |
| *Cxcl9* | chemokine (C-X-C motif) ligand 9 | 5.14858 | 2.82E-08 | 1.60E-07 |
| *Il17a* | interleukin 17A | 5.04618 | 2.10E-09 | 4.34E-08 |
| *Ido1* | indoleamine 2,3-dioxygenase 1 | 5.0206 | 5.84E-08 | 2.73E-07 |
| *Blk* | B lymphoid kinase | 4.66588 | 8.02E-08 | 3.47E-07 |
| *Cd96* | CD96 antigen | 4.61099 | 3.69E-09 | 5.57E-08 |
| *Cxcl11* | chemokine (C-X-C motif) ligand 11 | 4.60387 | 2.69E-10 | 3.17E-08 |
| *Nkg7* | natural killer cell group 7 sequence | 4.44413 | 3.20E-08 | 1.73E-07 |
| *Gbp2b* | guanylate binding protein 2b | 4.38208 | 3.31E-07 | 1.17E-06 |
| *Oas3* | 2'-5' oligoadenylate synthetase 3 | 4.25493 | 1.24E-07 | 5.12E-07 |
| *H2-DMb2* | histocompatibility 2, class II, locus  Mb2 | 4.12145 | 1.73E-07 | 6.92E-07 |
| *Il2ra* | interleukin 2 receptor, alpha chain | 3.95641 | 8.49E-09 | 8.34E-08 |
| *Ctla4* | cytotoxic T-lymphocyte-associated  protein 4 | 3.9549 | 3.07E-07 | 1.10E-06 |
| *Gbp3* | guanylate binding protein 3 | 3.87241 | 3.79E-09 | 5.59E-08 |
| *Mmp7* | matrix metallopeptidase 7 | 3.81363 | 3.14E-07 | 1.11E-06 |
| *Klrk1* | killer cell lectin-like receptor subfamily  K, member 1 | 3.68065 | 3.39E-07 | 1.18E-06 |
| *Tnf* | tumor necrosis factor | 3.64758 | 3.08E-07 | 1.10E-06 |
| *H2-Eb1* | histocompatibility 2, class II antigen E  beta | 3.6223 | 2.05E-07 | 7.93E-07 |
| *Cxcr6* | chemokine (C-X-C motif) receptor 6 | 3.58559 | 3.72E-11 | 2.19E-08 |
| *Cd6* | CD6 antigen | 3.58245 | 9.22E-07 | 2.74E-06 |
| *Ifit1* | interferon-induced protein with  tetratricopeptide repeats 1 | 3.55037 | 7.22E-07 | 2.23E-06 |
| *Zap70* | zeta-chain (TCR) associated protein kinase | 3.53521 | 2.03E-05 | 4.46E-05 |
| *Socs1* | suppressor of cytokine signaling 1 | 3.50511 | 7.55E-09 | 7.81E-08 |
| *Nlrc5* | NLR family, CARD domain containing 5 | 3.49574 | 1.98E-08 | 1.29E-07 |
| *Cd5* | CD5 antigen | 3.42563 | 3.48E-05 | 7.35E-05 |
| *Cd3g* | CD3 antigen, gamma polypeptide | 3.41817 | 4.83E-08 | 2.33E-07 |
| *Isg15* | ISG15 ubiquitin-like modifier | 3.41154 | 3.06E-08 | 1.70E-07 |
| *Cd247* | CD247 antigen | 3.33701 | 2.56E-08 | 1.51E-07 |
| *H2-DMa* | histocompatibility 2, class II, locus  DMa | 3.33632 | 1.71E-09 | 4.20E-08 |
| *Il2rb* | interleukin 2 receptor, beta chain | 3.30487 | 1.52E-07 | 6.14E-07 |
| *Ccr5* | chemokine (C-C motif) receptor 5 | 3.26057 | 7.15E-08 | 3.17E-07 |
| *Tnfsf8* | tumor necrosis factor (ligand) superfamily, member 8 | 3.24273 | 3.18E-06 | 8.32E-06 |
| *Irf4* | interferon regulatory factor 4 | 3.22545 | 6.73E-06 | 1.65E-05 |
| *Pdcd1* | programmed cell death 1 | 3.18532 | 2.73E-06 | 7.24E-06 |
| *Cd74* | CD74 antigen (invariant polypeptide of major histocompatibility complex,  class II antigen-associated) | 3.18264 | 8.32E-10 | 3.66E-08 |

| *H2-Ab1* | histocompatibility 2, class II antigen A,  beta 1 | 3.17124 | 7.86E-10 | 3.66E-08 |
| --- | --- | --- | --- | --- |
| *Cd3e* | CD3 antigen, epsilon polypeptide | 3.16658 | 6.70E-07 | 2.11E-06 |
| *Ikzf3* | IKAROS family zinc finger 3 | 3.10956 | 3.85E-06 | 9.81E-06 |
| *Gbp2* | guanylate binding protein 2 | 3.10604 | 6.17E-08 | 2.80E-07 |
| *Ltb* | lymphotoxin B | 3.08763 | 2.90E-08 | 1.63E-07 |
| *H2-Aa* | histocompatibility 2, class II antigen A,  alpha | 3.0496 | 4.24E-09 | 5.95E-08 |
| *Cd3d* | CD3 antigen, delta polypeptide | 3.02073 | 1.98E-05 | 4.37E-05 |
| *Ccl5* | chemokine (C-C motif) ligand 5 | 3.00967 | 4.29E-06 | 1.08E-05 |
| *Stat1* | signal transducer and activator of  transcription 1 | 2.99529 | 1.87E-08 | 1.28E-07 |
| *Il18bp* | interleukin 18 binding protein | 2.93516 | 1.91E-10 | 3.08E-08 |
| *Cd40* | CD40 antigen | 2.91884 | 5.22E-07 | 1.69E-06 |
| *Psmb8* | proteasome (prosome, macropain) subunit, beta type 8 | 2.89424 | 1.22E-08 | 1.03E-07 |
| *Itgal* | integrin alpha L | 2.86333 | 1.85E-07 | 7.32E-07 |
| *Icos* | inducible T cell co-stimulator | 2.84967 | 8.84E-09 | 8.53E-08 |
| *Pvrig* | negative regulator of TCR | 2.84653 | 6.07E-09 | 7.16E-08 |
| *Cd4* | CD4 antigen | 2.83088 | 1.41E-09 | 3.66E-08 |
| *Ly9* | lymphocyte antigen 9 | 2.83022 | 4.01E-05 | 8.40E-05 |
| *Cd274* | CD274 antigen | 2.81169 | 1.73E-08 | 1.25E-07 |
| *Traf1* | TNF receptor-associated factor 1 | 2.8026 | 1.43E-05 | 3.26E-05 |
| *Il21r* | interleukin 21 receptor | 2.79845 | 5.46E-05 | 0.000111 |
| *Itgae* | integrin alpha E, epithelial-associated | 2.78364 | 1.39E-09 | 3.66E-08 |
| *Cd2* | CD2 antigen | 2.76553 | 1.03E-05 | 2.41E-05 |
| *Rsad2* | radical S-adenosyl methionine domain containing 2 | 2.74271 | 3.75E-07 | 1.27E-06 |
| *Herc6* | hect domain and RLD 6 | 2.74062 | 1.43E-08 | 1.11E-07 |
| *Ifit3* | interferon-induced protein with  tetratricopeptide repeats 3 | 2.73242 | 2.78E-08 | 1.59E-07 |
| *Cxcr5* | chemokine (C-X-C motif) receptor 5 | 2.71749 | 0.000369 | 0.000649 |
| *Cd79b* | CD79B antigen | 2.71549 | 0.000592 | 0.00101 |
| *Fcgr4* | Fc receptor, IgG, low affinity IV | 2.70764 | 1.31E-09 | 3.66E-08 |
| *Mmp13* | matrix metallopeptidase 13 | 2.69938 | 1.01E-06 | 2.99E-06 |
| *Tnfsf10* | tumor necrosis factor (ligand) superfamily, member 10 | 2.69211 | 1.33E-08 | 1.06E-07 |
| *Oas2* | 2'-5' oligoadenylate synthetase 2 | 2.65808 | 1.33E-08 | 1.06E-07 |
| *Lck* | lymphocyte protein tyrosine kinase | 2.65263 | 1.97E-08 | 1.29E-07 |
| *Il2rg* | interleukin 2 receptor, gamma chain | 2.64829 | 1.81E-08 | 1.26E-07 |
| *Cd19* | CD19 antigen | 2.64765 | 0.000446 | 0.000775 |
| *Btla* | B and T lymphocyte associated | 2.6438 | 5.94E-07 | 1.90E-06 |
| *Ccl8* | chemokine (C-C motif) ligand 8 | 2.60718 | 2.59E-08 | 1.51E-07 |
| *Ly6c1* | lymphocyte antigen 6 complex, locus  C1 | 2.5878 | 1.44E-10 | 3.08E-08 |
| *Cxcl13* | chemokine (C-X-C motif) ligand 13 | 2.56828 | 2.45E-07 | 9.21E-07 |
| *Fcer1a* | Fc receptor, IgE, high affinity I, alpha polypeptide | 2.54444 | 1.57E-08 | 1.20E-07 |
| *Spib* | Spi-B transcription factor | 2.49518 | 2.66E-07 | 9.81E-07 |
| *Cd84* | CD84 antigen | 2.49276 | 4.82E-08 | 2.33E-07 |
| *Slamf7* | SLAM family member 7 | 2.48826 | 1.22E-06 | 3.55E-06 |

| *Itgax* | integrin alpha X | 2.4797 | 1.78E-08 | 1.26E-07 |
| --- | --- | --- | --- | --- |
| *Tap1* | transporter 1, ATP-binding cassette,  sub-family B | 2.45716 | 1.20E-09 | 3.66E-08 |
| *Itgb2* | integrin beta 2 | 2.44146 | 1.95E-09 | 4.34E-08 |
| *Icam1* | intercellular adhesion molecule 1 | 2.43741 | 4.24E-09 | 5.95E-08 |
| *Ikzf1* | IKAROS family zinc finger 1 | 2.42949 | 0.000164 | 0.000304 |
| *Irf7* | interferon regulatory factor 7 | 2.41926 | 1.43E-08 | 1.11E-07 |
| *Ctss* | cathepsin S | 2.41819 | 4.74E-09 | 6.38E-08 |
| *Ifit2* | interferon-induced protein with  tetratricopeptide repeats 2 | 2.40378 | 7.38E-08 | 3.24E-07 |
| *Mmp12* | matrix metallopeptidase 12 | 2.38311 | 9.84E-09 | 9.20E-08 |
| *Cd48* | CD48 antigen | 2.35094 | 2.40E-07 | 9.12E-07 |
| *Ccl19* | chemokine (C-C motif) ligand 19 | 2.34813 | 0.000245 | 0.000438 |
| *H2-Ob* | histocompatibility 2, O region beta  locus | 2.32394 | 3.79E-06 | 9.71E-06 |
| *Itga4* | integrin alpha 4 | 2.31808 | 1.17E-05 | 2.71E-05 |
| *Csf2rb* | colony stimulating factor 2 receptor,  beta, low-affinity | 2.30104 | 1.19E-08 | 1.03E-07 |
| *Psmb9* | proteasome (prosome, macropain) subunit, beta type 9 | 2.29859 | 2.23E-09 | 4.34E-08 |
| *Ms4a6b* | membrane-spanning 4-domains, subfamily A, member 6B | 2.29739 | 3.34E-08 | 1.77E-07 |
| *Ms4a1* | membrane-spanning 4-domains, subfamily A, member 1 | 2.26237 | 0.000118 | 0.000224 |
| *Irf8* | interferon regulatory factor 8 | 2.2563 | 6.96E-09 | 7.46E-08 |
| *Lyz1* | lysozyme 1 | 2.24631 | 2.43E-09 | 4.34E-08 |
| *Tapbpl* | TAP binding protein-like | 2.24213 | 1.18E-05 | 2.72E-05 |
| *Il16* | interleukin 16 | 2.21346 | 2.70E-06 | 7.20E-06 |
| *Cybb* | cytochrome b-245, beta polypeptide | 2.19457 | 1.18E-08 | 1.03E-07 |
| *Pirb* | paired Ig-like receptor B | 2.17478 | 9.25E-10 | 3.66E-08 |
| *Trat1* | T cell receptor associated transmembrane adaptor 1 | 2.16509 | 9.43E-06 | 2.24E-05 |
| *Psmb10* | proteasome (prosome, macropain)  subunit, beta type 10 | 2.16451 | 7.87E-10 | 3.66E-08 |
| *Lag3* | lymphocyte-activation gene 3 | 2.15688 | 4.96E-08 | 2.38E-07 |
| *C2* | complement component 2 (within H- 2S) | 2.13538 | 3.38E-09 | 5.23E-08 |
| *Pik3cd* | phosphatidylinositol 3-kinase catalytic  delta polypeptide | 2.13131 | 4.24E-05 | 8.86E-05 |
| *Ccl2* | chemokine (C-C motif) ligand 2 | 2.1281 | 4.51E-08 | 2.25E-07 |
| *Cd200r1* | CD200 receptor 1 | 2.1205 | 2.10E-08 | 1.33E-07 |
| *Nffibie* | nuclear factor of kappa light  polypeptide gene enhancer in B cells  inhibitor, epsilon | 2.10395 | 2.99E-09 | 4.89E-08 |
| *Tmem173* | transmembrane protein 173 | 2.10349 | 9.57E-08 | 4.05E-07 |
| *Sell* | selectin, lymphocyte | 2.10165 | 0.000473 | 0.000814 |
| *Cd28* | CD28 antigen | 2.09505 | 4.39E-08 | 2.21E-07 |
| *Pla2g2a* | phospholipase A2, group IIA (platelets,  synovial fluid) | 2.08238 | 4.85E-05 | 0.000101 |
| *Tlr1* | toll-like receptor 1 | 2.08181 | 1.97E-08 | 1.29E-07 |
| *B2m* | beta-2 microglobulin | 2.08157 | 1.31E-09 | 3.66E-08 |
| *Irf1* | interferon regulatory factor 1 | 2.07679 | 6.65E-09 | 7.25E-08 |
| *Thy1* | thymus cell antigen 1, theta | 2.07409 | 2.97E-09 | 4.89E-08 |
| *Lair1* | leukocyte-associated Ig-like receptor 1 | 2.07362 | 1.20E-07 | 4.97E-07 |

| *Samsn1* | SAM domain, SH3 domain and nuclear  localization signals, 1 | 2.05797 | 1.08E-06 | 3.19E-06 |
| --- | --- | --- | --- | --- |
| *H2-M3* | histocompatibility 2, M region locus 3 | 2.03219 | 6.08E-09 | 7.16E-08 |
| *Runx3* | runt related transcription factor 3 | 2.02571 | 2.21E-08 | 1.37E-07 |
| *Ptprc* | protein tyrosine phosphatase,  receptor type, C | 2.01791 | 2.37E-06 | 6.36E-06 |
| *Itga2* | integrin alpha 2 | 2.00133 | 3.33E-08 | 1.77E-07 |
| *Gimap4* | GTPase, IMAP family member 4 | 1.99641 | 1.27E-06 | 3.66E-06 |
| *Lilrb4a* | leukocyte immunoglobulin-like  receptor, subfamily B, member 4A | 1.96261 | 3.19E-08 | 1.73E-07 |
| *Pik3r5* | phosphoinositide-3-kinase, regulatory subunit 5, p101 | 1.95337 | 8.12E-06 | 1.97E-05 |
| *Tap2* | transporter 2, ATP-binding cassette,  sub-family B (MDR/TAP) | 1.94496 | 3.23E-09 | 5.14E-08 |
| *Ccr2* | chemokine (C-C motif) receptor 2 | 1.94494 | 3.02E-07 | 1.09E-06 |
| *Ccl9* | chemokine (C-C motif) ligand 9 | 1.89179 | 2.36E-08 | 1.43E-07 |
| *Vcam1* | vascular cell adhesion molecule 1 | 1.89107 | 1.06E-08 | 9.61E-08 |
| *H2-T23* | histocompatibility 2, T region locus 23 | 1.87613 | 3.36E-06 | 8.75E-06 |
| *Clec7a* | C-type lectin domain family 7,  member a | 1.83989 | 5.93E-09 | 7.16E-08 |
| *Cd86* | CD86 antigen | 1.8273 | 1.62E-08 | 1.21E-07 |
| *Il7r* | interleukin 7 receptor | 1.82282 | 3.49E-07 | 1.21E-06 |
| *H2-Q2* | histocompatibility 2, Q region locus 2 | 1.81948 | 3.01E-06 | 7.91E-06 |
| *Cd300a* | CD300A antigen | 1.81248 | 6.14E-08 | 2.80E-07 |
| *Ccl7* | chemokine (C-C motif) ligand 7 | 1.80896 | 2.14E-06 | 5.82E-06 |
| *Ccl12* | chemokine (C-C motif) ligand 12 | 1.80431 | 1.85E-06 | 5.18E-06 |
| *Casp1* | caspase 1 | 1.80109 | 1.91E-07 | 7.45E-07 |
| *Il1b* | interleukin 1 beta | 1.79442 | 4.91E-05 | 0.000101 |
| *C1qb* | complement component 1, q  subcomponent, beta polypeptide | 1.79112 | 1.16E-09 | 3.66E-08 |
| *C1qa* | complement component 1, q  subcomponent, alpha polypeptide | 1.79046 | 1.05E-09 | 3.66E-08 |
| *Tnfrsf14* | tumor necrosis factor receptor superfamily, member 14 | 1.78609 | 2.37E-09 | 4.34E-08 |
| *Mx1* | MX dynamin-like GTPase 1 | 1.77843 | 2.30E-08 | 1.41E-07 |
| *Ccl22* | chemokine (C-C motif) ligand 22 | 1.77485 | 0.000101 | 0.000195 |
| *Ifi203* | interferon activated gene 203 | 1.77136 | 7.41E-07 | 2.27E-06 |
| *Adgre1* | adhesion G protein-coupled receptor E1 | 1.75439 | 2.48E-08 | 1.48E-07 |
| *Fcgr1* | Fc receptor, IgG, high affinity I | 1.74729 | 7.92E-10 | 3.66E-08 |
| *Gimap6* | GTPase, IMAP family member 6 | 1.72974 | 1.79E-07 | 7.11E-07 |
| *Stat2* | signal transducer and activator of transcription 2 | 1.72858 | 1.28E-08 | 1.06E-07 |
| *Ifitm1* | interferon induced transmembrane protein 1 | 1.70479 | 8.09E-08 | 3.48E-07 |
| *Hck* | hemopoietic cell kinase | 1.69421 | 1.62E-08 | 1.21E-07 |
| *Parp9* | poly (ADP-ribose) polymerase family,  member 9 | 1.68225 | 1.74E-08 | 1.25E-07 |
| *Nfam1* | Nfat activating molecule with ITAM motif 1 | 1.67894 | 8.00E-05 | 0.000158 |
| *P2ry13* | purinergic receptor P2Y, G-protein coupled 13 | 1.67668 | 3.83E-08 | 1.97E-07 |
| *H2-D1* | histocompatibility 2, D region locus 1 | 1.66729 | 6.68E-10 | 3.66E-08 |

| *Parp12* | poly (ADP-ribose) polymerase family, member 12 | 1.6442 | 2.45E-08 | 1.47E-07 |
| --- | --- | --- | --- | --- |
| *Gpsm3* | G-protein signalling modulator 3  (AGS3-like, C. elegans) | 1.64138 | 2.48E-07 | 9.23E-07 |
| *Dll1* | delta-like 1 (Drosophila) | 1.63568 | 1.02E-08 | 9.37E-08 |
| *Cd80* | CD80 antigen | 1.63072 | 4.81E-07 | 1.57E-06 |
| *Fcgr3* | Fc receptor, IgG, low affinity III | 1.62849 | 4.12E-07 | 1.38E-06 |
| *Dtx3l* | deltex 3-like (Drosophila) | 1.6102 | 8.35E-09 | 8.34E-08 |
| *Fcgr2b* | Fc receptor, IgG, low affinity IIb | 1.59354 | 7.19E-09 | 7.56E-08 |

**Supplemental Table 4.** Pathways which are significantly impacted in the *InsGAS^tg/tg^Il17ra^-/-^* mice based on gene expression profiling using the PanCancer IO 360 Panel of 770 genes (Supplemental Table 3).

| **Panther**  **Term ID** | **Term Name** | | **p-Value** | | **FDR p-Adj** | | **# of Genes**  **in Term** | | **# of Genes that are**  **also in this panel** | | **# of Up-**  **regulated genes** | |
| --- | --- | --- | --- | --- | --- | --- | --- | --- | --- | --- | --- | --- |
| P00053 | T cell activation | | 6.50E-05 | | 0.00187 | | 70 | | 13 | | 13 | |
| **BioPlanet Term ID** | **Term Name** | | **p-Value** | | **FDR- p-Adj** | | **# of Genes**  **in Term** | | | **# of Genes that are**  **also in this panel** | | **# of Up-**  **regulated genes** |
| 894 | Adaptive immune  system | | 0 | | 0 | | 537 | | | 58 | | 58 |
| 905 | Immune system | | 1.00E-13 | | 2.20E-11 | | 883 | | | 86 | | 86 |
| 329 | T helper cell surface  molecules | | 6.30E-08 | | 8.90E-06 | | 13 | | | 12 | | 12 |
| 302 | Systemic lupus  erythematosus | | 1.00E-07 | | 1.10E-05 | | 125 | | | 18 | | 18 |
| 898 | Generation of second  messenger molecules | | 1.50E-07 | | 1.30E-05 | | 24 | | | 10 | | 10 |
| 238 | Cell adhesion  molecules (CAMs) | | 1.90E-07 | | 1.40E-05 | | 131 | | | 28 | | 28 |
| 611 | Interferon signaling | | 3.20E-07 | | 1.90E-05 | | 165 | | | 27 | | 27 |
| 464 | Antigen processing  and presentation | | 8.00E-07 | | 4.20E-05 | | 82 | | | 19 | | 19 |
| 901 | Costimulation by the  CD28 family | | 1.20E-06 | | 5.90E-05 | | 58 | | | 18 | | 18 |
| 204 | Phagosome | | 2.20E-06 | | 9.20E-05 | | 156 | | | 21 | | 21 |
| **Reactome Term ID** | | **Term Name** | | **p-Value** | | **FDR-**  **p-Adj** | | **# of Genes**  **in Term** | | **# of Genes that are**  **also in this panel** | | **# of Up-**  **regulated genes** |
| R-MMU- 198933 | | Immunoregulatory interactions (Lymphoid  and a non-Lymphoid cell) | | 4.20E-06 | | 0.00033 | | 96 | | 20 | | 20 |
| R-MMU-  202433 | | Generation of second  messenger molecules | | 8.00E-06 | | 0.00033 | | 20 | | 7 | | 7 |
| R-MMU-  202427 | | Phosphorylation of CD3  and TCR zeta chains | | 8.00E-06 | | 0.00033 | | 12 | | 7 | | 7 |
| R-MMU- 202430 | | Translocation of ZAP-70 to Immunological  synapse | | 8.00E-06 | | 0.00033 | | 9 | | 7 | | 7 |
| R-MMU-  389948 | | PD-1 signaling | | 4.60E-05 | | 0.00152 | | 13 | | 8 | | 8 |
| R-MMU-  2029481 | | FCGR activation | | 0.00088 | | 0.02368 | | 13 | | 6 | | 6 |
| R-MMU- 2029482 | | Regulation of actin  dynamics for phagocytic cup formation | | 0.00121 | | 0.02792 | | 57 | | 5 | | 5 |
| R-MMU-  202424 | | Downstream TCR  signaling | | 0.00369 | | 0.06579 | | 83 | | 10 | | 10 |
| R-MMU- 983170 | | Antigen Presentation: Folding, assembly and peptide loading of class I  MHC | | 0.00469 | | 0.06579 | | 41 | | 6 | | 6 |
| R-MMU-  1236974 | | ER-Phagosome pathway | | 0.00469 | | 0.06579 | | 34 | | 6 | | 6 |
